# Transmitting silks of maize have a complex and dynamic microbiome

**DOI:** 10.1038/s41598-021-92648-4

**Published:** 2021-06-24

**Authors:** Eman M. Khalaf, Anuja Shrestha, Jeffrey Rinne, Michael D. J. Lynch, Charles R. Shearer, Victor Limay-Rios, Lana M. Reid, Manish N. Raizada

**Affiliations:** 1 Department of Plant Agriculture, University of Guelph, 50 Stone Road E, Guelph, ON N1G 2W1 Canada; 2 Department of Microbiology and Immunology, Faculty of Pharmacy, Damanhour University, Damanhour, 22511 Egypt; 3 Metagenom Bio, 550 Parkside Drive, Unit A9, Waterloo, ON N2L 5V4 Canada; 4 Department of Plant Agriculture, University of Guelph, Ridgetown Campus, 120 Main Street E, Ridgetown, ON N0P 2C0 Canada; 5 Ottawa Research and Development Centre, Agriculture and Agri-Food Canada, 960 Carling Avenue, Central Experimental Farm, Ottawa, ON K1A 0C6 Canada

**Keywords:** Plant sciences, Plant immunity, Microbial communities, Fungal pathogenesis

## Abstract

In corn/maize, silks emerging from cobs capture pollen, and transmit resident sperm nuclei to eggs. There are > 20 million silks per U.S. maize acre. Fungal pathogens invade developing grain using silk channels, including *Fusarium graminearum* (*Fg*, temperate environments) and devastating carcinogen-producers (Africa/tropics). *Fg* contaminates cereal grains with mycotoxins, in particular Deoxynivalenol (DON), known for adverse health effects on humans and livestock. Fitness selection should promote defensive/healthy silks. Here, we report that maize silks, known as styles in other plants, possess complex and dynamic microbiomes at the critical pollen-fungal transmission interval (henceforth: transmitting style microbiome, TSM). Diverse maize genotypes were field-grown in two trial years. MiSeq 16S rRNA gene sequencing of 328 open-pollinated silk samples (healthy/*Fg*-infected) revealed that the TSM contains > 5000 taxa spanning the prokaryotic tree of life (47 phyla/1300 genera), including nitrogen-fixers. The TSM of silk tip tissue displayed seasonal responsiveness, but possessed a reproducible core of 7–11 MiSeq-amplicon sequence variants (ASVs) dominated by a single *Pantoea* MiSeq-taxon (15–26% of sequence-counts). *Fg*-infection collapsed TSM diversity and disturbed predicted metabolic functionality, but doubled overall microbiome size/counts, primarily by elevating 7–25 MiSeq-ASVs, suggestive of a selective microbiome response against infection. This study establishes the maize silk as a model for fundamental/applied research of plant reproductive microbiomes.

## Introduction

In both plants and humans, reproduction requires maternal exposure to the environment to facilitate sperm entry (female reproductive tract), thereby also creating an entry point for pathogens^1,2^. In humans, disruptions in the microbiomes of the vaginal-cervical-uterine-Fallopian tube tract have been associated with disease, infertility and pre-term birth^3,4^. In plants, the female reproductive tract includes exposed papillae (stigma), on which pollen land and germinate, connected to a long channel (style) that terminates in an egg sac; sperm nuclei exit the pollen, then travel to the egg within a tubular extension of the pollen (pollen tube) inside the style channel^5^. There are limited reports of cultured microbes and/or microbiomes associated with the stigma and style from a few plants, including pear^6^, the wild tree *Metrosideros polymorpha*^7^, wild monkeyflowers^8^ and apples^9^.

In cultivated maize (corn, *Zea mays* L.), one of the world’s three most important food crops, the style is called the silk, recognizable as the threads that emerge at the tips of corn cobs. The maize silk is one of the fastest-growing tissues in nature (~ 1–3 mm/h)^10^. Due to climate change, silk health is becoming more critical to global maize production, because drought inhibits silk growth which prevents synchronization between pollen shed and silk receptivity and thus reduces grain yield^11^. Silk emergence is also susceptible to low soil nitrogen^12^, which is particularly limiting in Africa where maize is a staple food crop. Silks have been used for centuries in Mexico, China and elsewhere as herbal medicines for diseases of the urinary tract, arthritis, obesity, and for their anti-microbial activities, associated with a diversity of phytochemicals^13^.

Silks are also critically important to the world’s maize farmers not only for seed establishment but also because plant insects (including the maize earworm, *Helicoverpa zea*)^14^ and pathogens utilize silks as express entry routes to the developing grain^2^ analogous to *Candida* invading the vagina^15^. After fertilization, silks become senescent but remain rich in nutrients^2,13^, and thus this stage of silk development is particularly susceptible to grain-bound pathogens^2^. In tropical Africa and Latin America, silk-invaders include the mycotoxin-producing fungal pathogens *Aspergillus flavus* and *Fusarium verticillioides* that devastate tens of millions of maize farmers, where they produce carcinogens^2,16^. In primarily temperate regions, silk-invading *F. graminearum* (*Fg*) infection causes Gibberella ear rot^16^. *Fg* produces mycotoxins, mainly type B trichothecenes [including deoxynivalenol (DON) and nivalenol (NIV)] and zearalenone (ZEA)—reported for their toxic impacts on gastrointestinal, hematic and reproductive systems, respectively, which impact livestock and human health^16^. Altered temperature, rainfall, and humidity associated with climate change are expected to increase *Fg*-based mycotoxins in maize^17^.

Despite the importance of styles/silks to both reproduction and global grain disease, there are no reports in the literature about the microbiome of silks. Maize silks are environmentally exposed and unusually long (up to 30 cm)^5^, and hence an excellent model for style microbiome research. Silks would be an ideal habitat for microbes, as they are rich in lipids, proteins, carbohydrates, minerals, and vitamins, and have a high moisture content^13^. Endophytic microbes inhabiting other tissues in maize and its relatives have been shown to combat *Fg*^18–22^. There may have been even stronger selection pressure to recruit protective microbes within the actual entry/transmission point of *Fg* and other pathogens/pests, as well as additional microbes to protect the tissue against environmental stress (e.g. drought, nutrients), given its critical importance to reproduction. However, the rapid growth rate of silk tissue and its exposure to a fluctuating environment raise questions as to whether silks/styles can establish a microbiome and whether it would be stable.

Here, we test: (1) whether maize silks at the pollen-fungal transmission interval possess core microbiomes within the spatial tip to base gradient; (2) whether these microbiomes are stable across field spatial blocks, trial years and diverse host genotypes; and (3) whether *Fg* invasion using artificial spore inoculation disrupts the transmitting silk microbiome and/or elicits increases in potential protective microbes (*Fg*-indicator taxa). Here, the microbiome was analyzed in open pollinated silks to mimic maize production in farmers’ fields after pollination when *Fg* primarily enters. We henceforth refer to this as the transmitting style microbiome (TSM), the microbiome associated with silks at the interval encompassing pollination and *Fg* transmission.

## Results

### Exploration of the healthy transmitting silk microbiome (TSM)

**Figure 1:**
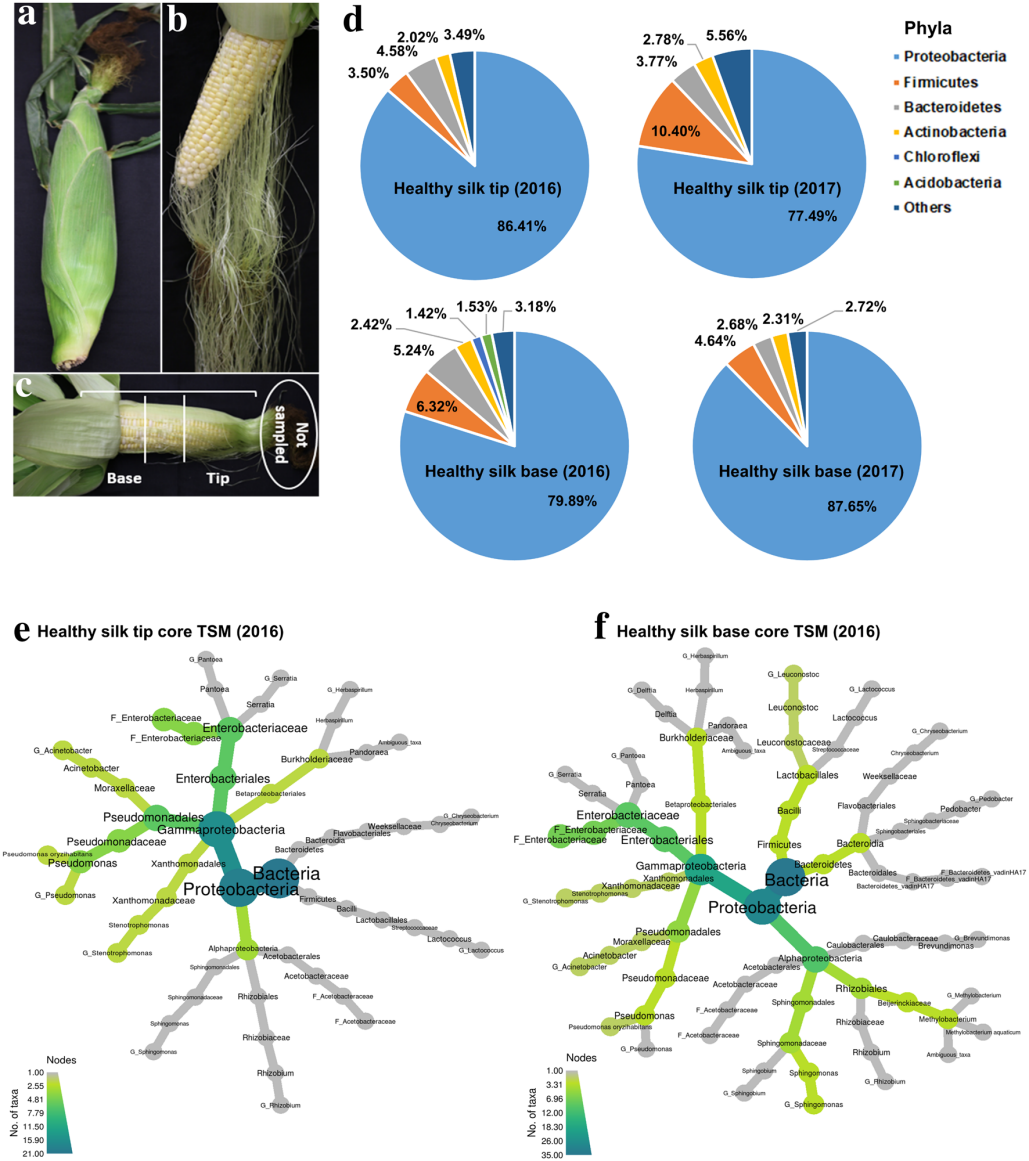
Composition of the transmissible style microbiome (TSM) of healthy maize silks. (**a**) Photo of maize cob with husk leaves protecting silks; (**b**) Peeled away husk leaves revealing/exposing silk threads; (**c**) Locations of base and tip silk tissues used in this study. (**d**) Contribution of dominant bacterial phyla in healthy TSM based on calculated relative abundance (RA) ≥ 1% using 16S read counts. (**e**, **f**) Heat tree displays of the core microbiomes of healthy transmitting silks harvested in 2016 from the (**e**) silk tip and (**f**) silk base (see Supplementary Fig. S5c, e for 2017). Core taxa (prevalent ≥ 50% of silk samples) are displayed in a hierarchal taxonomic heat tree from kingdom to species. The color depth and node size indicate the number of bacterial taxa within each taxonomic node or branch.

To determine whether healthy transmitting silks have a stable microbiome, 14 diverse maize genotypes (primarily exhibiting some degree of silk resistance to *Fg*), spanning 7 heterotic breeding groups (Supplementary Table S1), were grown in 3 spatial field blocks for 2 years (weather data: Supplementary Figs. S1, S2). Silks were harvested from 3 randomly selected cobs per block and pooled, with husk leaf-protected silk tip and base tissues first separated (Fig. 1a–c), resulting in 163 genomic DNA samples. After quality filtering, there were ~ 3 million 16S rRNA (V4 region) read counts (Supplementary Table S2). Fastq files of obtained sequences were deposited in Genbank under the BioProject accession no. PRJNA601168. Silk tip genomic DNA isolation was prioritized, using a DNeasy Plant Mini kit (Qiagen, USA), which resulted in sufficient yields for MiSeq; however subsequent silk base DNA isolations showed low yields using this method, causing us to adapt a higher-yielding CTAB protocol with more starting tissue. When tested on the same split samples (as a control), the Qiagen kit method was observed to produce greater microbial diversity than the CTAB method (Supplementary Fig. S3). Therefore, for this study, we have bioinformatically analyzed and presented each tissue location (tip vs base) separately and avoided direct comparisons. In general, the silk base tissue results may be underestimating microbial diversity.

In both tip and base silk tissues, the most dominant bacterial phylum was *Proteobacteria* (which occupied ~ 78–88% of all 16S read counts in both 2016 and 2017 based on relative abundance, RA), followed far behind by *Firmicutes*, *Bacteroidetes* and *Actinobacteria* (RA > 1%) (Fig. 1d). Within the *Proteobacteria*, the most dominant class, order and genus, respectively, were *Gammaproteobacteria*, *Enterobacteriales* and *Pantoea* (Table 1, Supplementary Table S3a, b). Healthy silks, when combined, were inhabited by 5152 unique bacterial (99.8%) and archaea taxa (0.2%) belonging to 1296 genera spanning the Prokaryotic Tree of Life^23^ (Supplementary Fig. S4). The results showed the existence of reproducible core tip and base TSM (taxa prevalent in at least 50% of samples) traversing the maize genotypes and field years (Fig. 1e, f; Supplementary Fig. S5c, e, Supplementary Fig. S6). There were surprising TSM inhabitants such as widespread nitrogen-fixing bacteria including *Rhizobium, Herbaspirillum* and *Azospirillum* (Table 1, Fig. 1e, f, Supplementary Fig. S6a–d).

**Table 1:** Summary of TSM taxa at genus level inhabiting silk tip tissues with mean relative abundance of > 1% in at least one of the 4 sample groups.

Taxonomic level	Taxon	Healthy tip (2016)	*Fg*-infected tip (2016)	Healthy tip (2017)	*Fg*-infected tip (2017)
Genus		N = 455	N = 214	N = 531	N = 311
	Pantoea	24.47	21.63	24.58	43.49
	Acinetobacter	14.58	20.71	6.63	7.06
	Pseudomonas	10.81	13.18	9.21	11.38
	Serratia	6.09	6.73	< 1.0	< 1.0
	Stenotrophomonas	5.81	10.77	5.88	12.47
	Sphingomonas	4.82	5.98	3.09	2.55
	Chryseobacterium	3.06	3.34	1.39	1.51
	Pandoraea	2.05	2.00	< 1.0	< 1.0
	Ambiguous_taxa	1.95	2.48	< 1.0	< 1.0
	Herbaspirillum	1.6	2.08	< 1.0	1.74
	Lactococcus	1.26	< 1.0	3.36	3.42
	Sphingobacterium	1.03	1.25	1.09	2.52
	Leuconostoc	1.01	< 1.0	< 1.0	< 1.0
	Acetobacter	< 1.0	1.87	< 1.0	< 1.0
	Rhizobium*	< 1.0	1.29	< 1.0	1.63
	Clostridium ss 8**	< 1.0	< 1.0	1.04	< 1.0
	Enterococcus	< 1.0	< 1.0	1.89	< 1.0
	Escherichia-Shigella	< 1.0	< 1.0	1.29	< 1.0
	Exiguobacterium	< 1.0	< 1.0	1.05	< 1.0
	Gluconobacter	< 1.0	< 1.0	1.81	< 1.0
	Lactobacillus	< 1.0	< 1.0	1.49	< 1.0
	Massilia	< 1.0	< 1.0	1.27	< 1.0
	Phyllobacterium	< 1.0	< 1.0	1.75	< 1.0
	Delftia	< 1.0	< 1.0	< 1.0	3.31
Taxa		N = 1580	N = 813	N = 1466	N = 1022

*Allorhizobium-Neorhizobium-Pararhizobium-Rhizobium.

**Clostridium sensu stricto 8.

### Trial year impacts on the healthy transmitting silk microbiome

**Figure 2:**
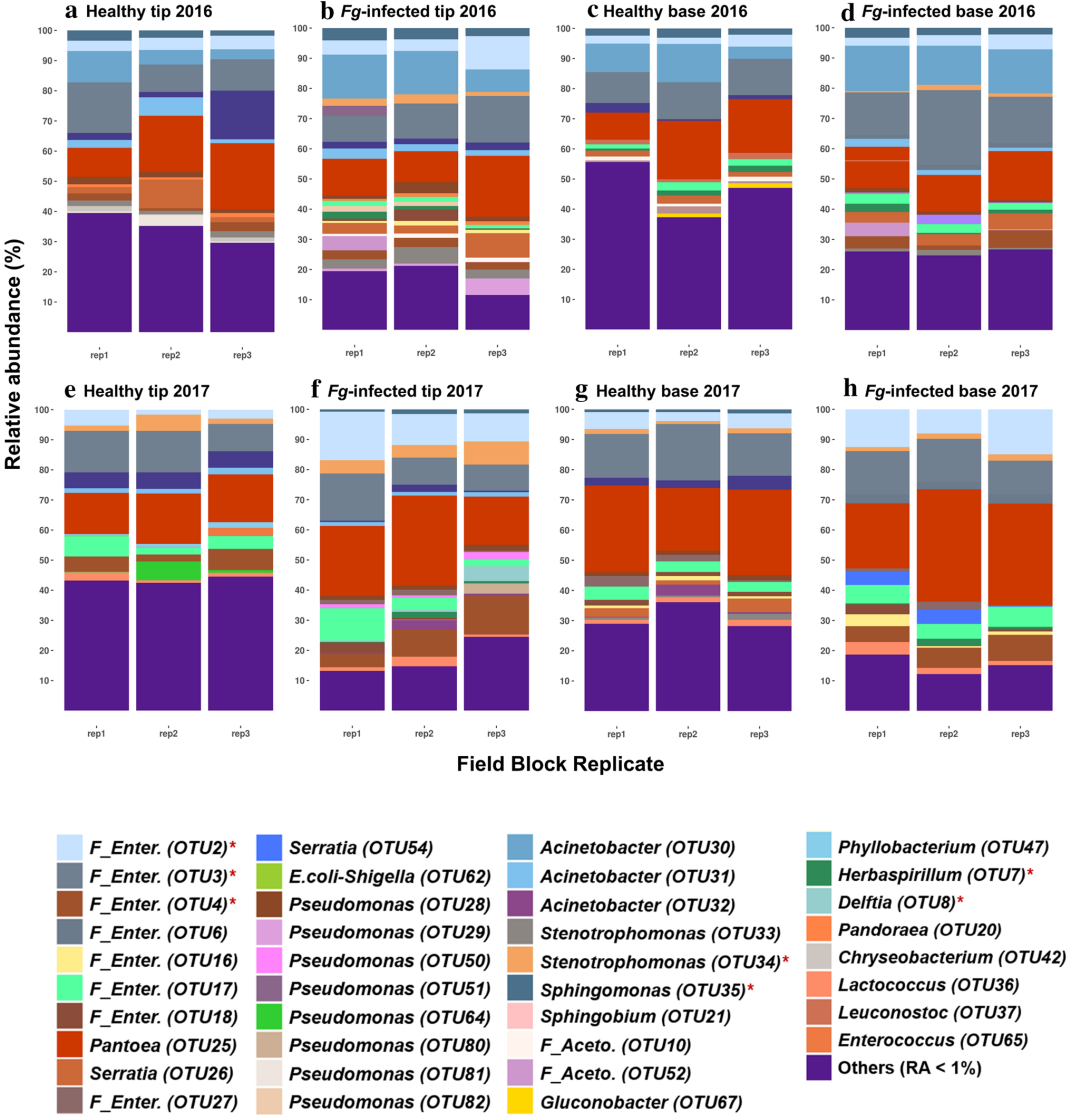
Impacts of field block location, year and *Fg* infection on TSM composition. (**a**–**h**) Bar charts of dominant TSM taxa (relative abundance (RA) ≥ 1%) at 3 field blocks (rep1, rep2, rep3) calculated at the OTU level for healthy transmitting silks in 2016 of (**a**) tip and (**c**) base tissues, and 2017 of (**e**) tip and (**g**) base tissues, and *Fg*-infected silks in 2016 of (**b**) tip and (**d**) base tissues, and 2017 of (**f**) tip and (**h**) base tissues. OTUs with RA < 1% were grouped and labeled as “Others”. Taxa tagged by a red asterisk denote consistent *Fg*-indicators.

The relative abundance of the dominant taxa was consistent across years (Table 1; Supplementary Table S3a, b; Fig. 2a, c, e, g) providing evidence for the stability of dominant TSM taxa (e.g. including *Pantoea* and *Acinetobacter* OTUs). This stability was confirmed by generally non-significant differences in estimates of alpha diversity indices across the different spatial field blocks across 2016 and 2017, with only two exceptions for observed OTUs and Faith’s phylogenetic diversity metric in healthy base tissues in 2017 (Supplementary Table S4). When these two independent statistical measurements are combined, they suggest that the transmitting female reproductive tract of modern maize is inhabited by a complex microbiome from which the dominant taxa are reproducible.

**Figure 3:**
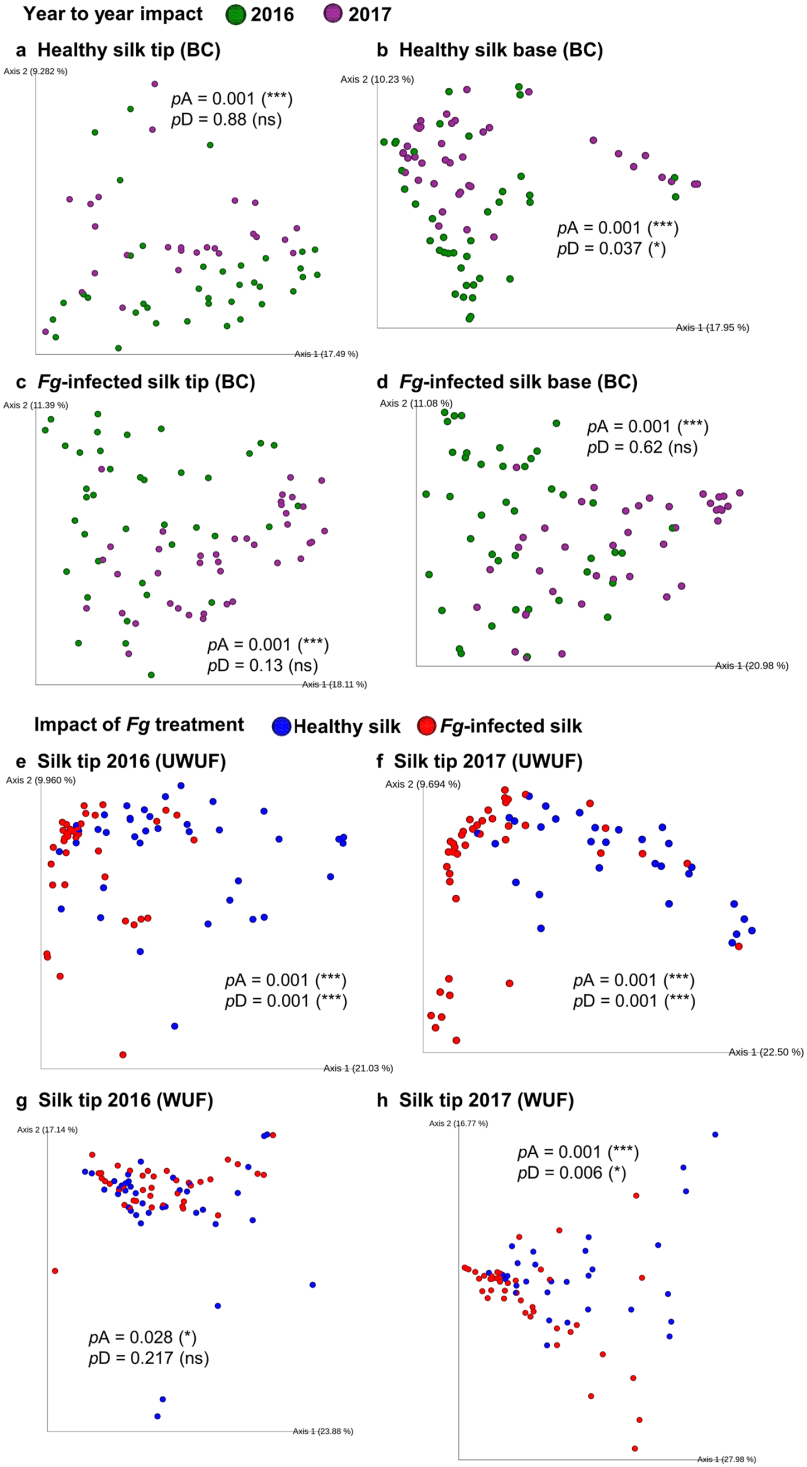
Principal Coordinate Plots (PCoA) of Beta diversity analysis. (**a**–**d**) 2D PCoA plots that display year-to-year TSM shifts using Bray–Curtis (BC) distance matrix: respectively, (**a**, **b**) represent healthy tip and base, and (**c**, **d**) represent *Fg*-infected tip and base (see Supplementary Fig. S7 (a–l) for other matrices where PCoA plots displayed as 5 principal coordinates (vertical lines) and each horizontal coloured line represents one silk sample), (**e**–**h**) 2D PCoA plots that display TSM shifts upon *Fg* infection using phylogeny-based distance matrices: (**e**, **f**) unweighted UniFrac (UWUF) that focuses on rare taxa in (**e**) tip tissues (2016), and (**f**) tip tissues (2017), and (**g**, **h**) weighted UniFrac (WUF) that focuses on dominant taxa in (**g**) tip tissues (2016), (**h**) tip tissues (2017) (see Supplementary Fig. S11 for other matrices and silk tissues displayed as 5 principal coordinates). *p*A, and *p*D denote the calculated *p* values of PERMANOVA, and PERMDISP tests, respectively (see Supplementary Table S5).

Despite the reproducibility of dominant taxa, a test of beta diversity showed a significant microbiome shift in healthy transmitting silk tips from 2016 to 2017 which could be statistically confirmed to be a trial year effect; this conclusion was less certain for base tissues (Supplementary Table S5a). These results were based on the PERMANOVA (permutational multivariate analysis of variance) test performed using the Bray–Curtis dissimilarity matrix (which provides 16S read count-abundance based measurements of dissimilarity across tested groups), unweighted UniFrac matrix [focuses on rarely shared (less abundant) taxa], and weighted UniFrac matrix (focused on dominant taxa, incorporating both phylogenetic distances along with abundances), followed by validation using the PERMDISP test which confirmed that the within-group dispersion of the data was not significantly different between the years, especially for tip samples (Supplementary Table S5a). The dissimilarities between tested sample groups were visualized by 2D principal coordinate analysis (PCoA) plots. The PCoA plots showed a greater shift in the tip TSM between years compared to base tissues (Fig. 3a, b; Supplementary Fig. S7a–l). Consistent with this result, there was a reduction in the diversity observed in the tip core microbiome in 2017 compared to 2016 (from 21 to 11 taxa) whereas the base was more stable (from 35 to 31 taxa) (Fig. 1e, f, and Supplementary Fig. S5a–f).

### Global impacts of *F. graminearum* (*Fg*) infection on the transmitting silk microbiome

To determine the impacts of *Fg* infection on the TSM, split-plots of all healthy plots (14 maize genotypes, 3 field blocks, 2 years) were artificially inoculated with *Fg* spores directly onto emerged silks, and then 165 treated samples (tip and base) were analyzed. Surprisingly, silk Fg infection led to a large increase in 16S read counts (from ~ 3 million in healthy transmitting silks to ~ 7 million in infected silks), yet dramatic reductions in the total number of taxa (from 5152 in healthy tissues to 3074 in Fg-infected tissues) (Table S2) confirmed by alpha diversity analysis (healthy vs Fg-infected group significance) (Table S4). The reduction was primarily associated with depletion of rare taxa (< 1% relative abundance) (Fig. S8). At the phyla level, *Proteobacteria* (especially *Gammaproteobacteria*) significantly increased in relative abundance while the remaining dominant phyla decreased (Supplementary Figs. S8–S10; Supplementary Dataset S1). These estimates were based on calculations performed in STAMP using 2-sided White’s non-parametric t-test adjusted for 2-group comparisons with a Benjamini–Hochberg multiple test correction method using collapsed feature tables (Supplementary Dataset S1).

The corresponding PCoA plots of UniFrac matrices confirmed the contraction in the diversity of the tip TSM upon *Fg*-infection [Supplementary Fig. S11, unweighted UniFrac that places equal weight on all taxa including rare taxa (defined here as RA < 1%)] and the significant increase in abundance of specific taxa primarily belonging to the phylum *Proteobacteria* [Supplementary Figs. S9, S10, weighted UniFrac matrix that focuses on dominant taxa (defined here as RA > 1%)]. These conclusions also were true in base tissues in both years, as confirmed using Bray–Curtis in particular (Supplementary Table S5b, Supplementary Fig. S12).

In beta diversity analysis, *Fg* was a greater driver of TSM composition compared to other factors including host genotype (Supplementary Fig. S7m–o). PERMANOVA suggested a significant shift in TSM composition upon *Fg* infection (Fig. 3e–h, Supplementary Fig. S11). However, there were often significant differences in the level of dispersion between treatment groups (PERMDISP, Supplementary Table S5b), consistent with the dramatic loss of diversity upon *Fg* infection, which when combined with conserved dominant taxa (Table 1; Fig. 2), caused overlap among the treatment groups in PCoA plots (Fig. 3e–h). The *Fg*-treated TSM was more sensitive to trial year compared to the TSM from healthy tissues (Fig. 3a–d, Supplementary Fig. S7a–l, Supplementary Table S5).

Together these results show that *Fg*-infection dramatically reduced overall diversity of the TSM (Supplementary Figs. S8, S12), yet conversely caused a reproducible proliferation of the total microbial population, associated with a small set of core dominant taxa that is resilient to *Fg*.

### Conservation and resiliency of core microbiomes

**Figure 4:**
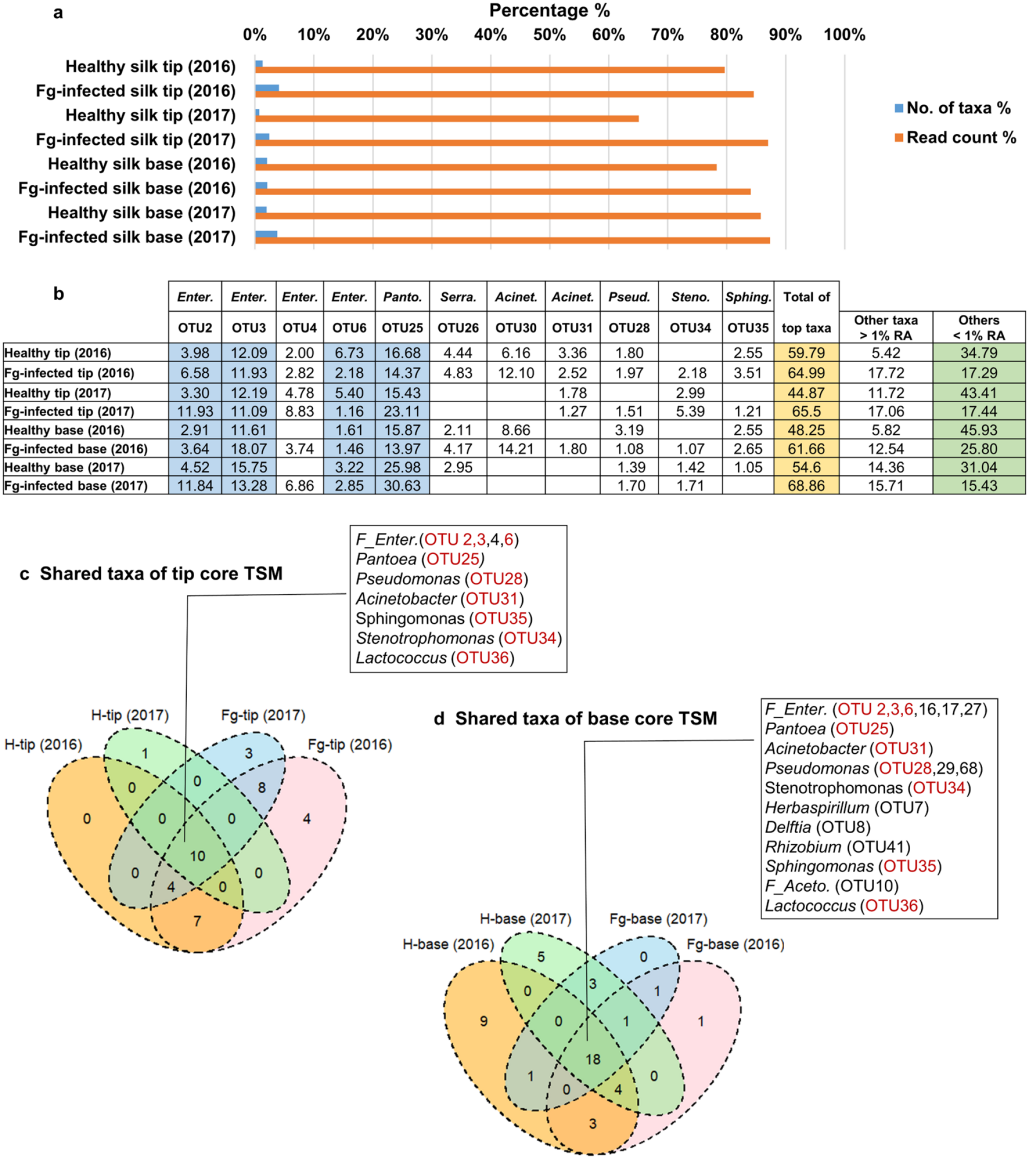
Dominance and prevalence of the core TSM. (**a**) Bar chart representing the percent contribution of the core microbiomes to their corresponding microbial communities. (**b**) Mean relative abundance (RA) of the most dominant and prevalent TSM taxa. (**c**, **d**) Venn diagrams of the shared taxa of core TSM: respectively, (**c**) tip core TSM, and (**d**) base core TSM.

Overall, the higher-level TSM taxonomic structure, dominated by the core taxa, was resilient to *Fg* infection across tissues and trial years (Figs. 2a–h, 3g, h; Supplementary Figs. S8, S9). Regardless of treatment, tissue location and year, and based on abundance, the core taxa comprised 0.75–4% of the total taxa count but contributed 65–87% of the total read count of each microbiome (Fig. 4a). *Fg* infection increased the number of taxa that comprised the tip core microbiome, but decreased the diversity of the base core microbiome, compared to healthy transmitting silks (Fig. 1e, f; Supplementary Fig. S5). Nine abundant OTUs (defined as the shared core TSM) were highly conserved (in at least 50% of the samples) in both healthy and *Fg*-infected silk tip and base tissues across years belonging to three bacterial classes: *Gammaproteobacteria* [*Pantoea* (OTU25), *Enterobacteriaceae* family (OTU2, OTU3, OTU6), *Pseudomonas* (OTU28), *Stenotrophomonas* (OTU34) and *Acinetobacter* (OTU31)], *Alphaproteobacteria* (*Sphingomonas,* OTU35) and *Bacilli* (*Lactococcus,* OTU36) (Fig. 4b–d). Of these, *Pantoea* (OTU25) was the most prevalent taxon, detected in almost all healthy and infected samples (327/328 samples).

**Figure 5:**
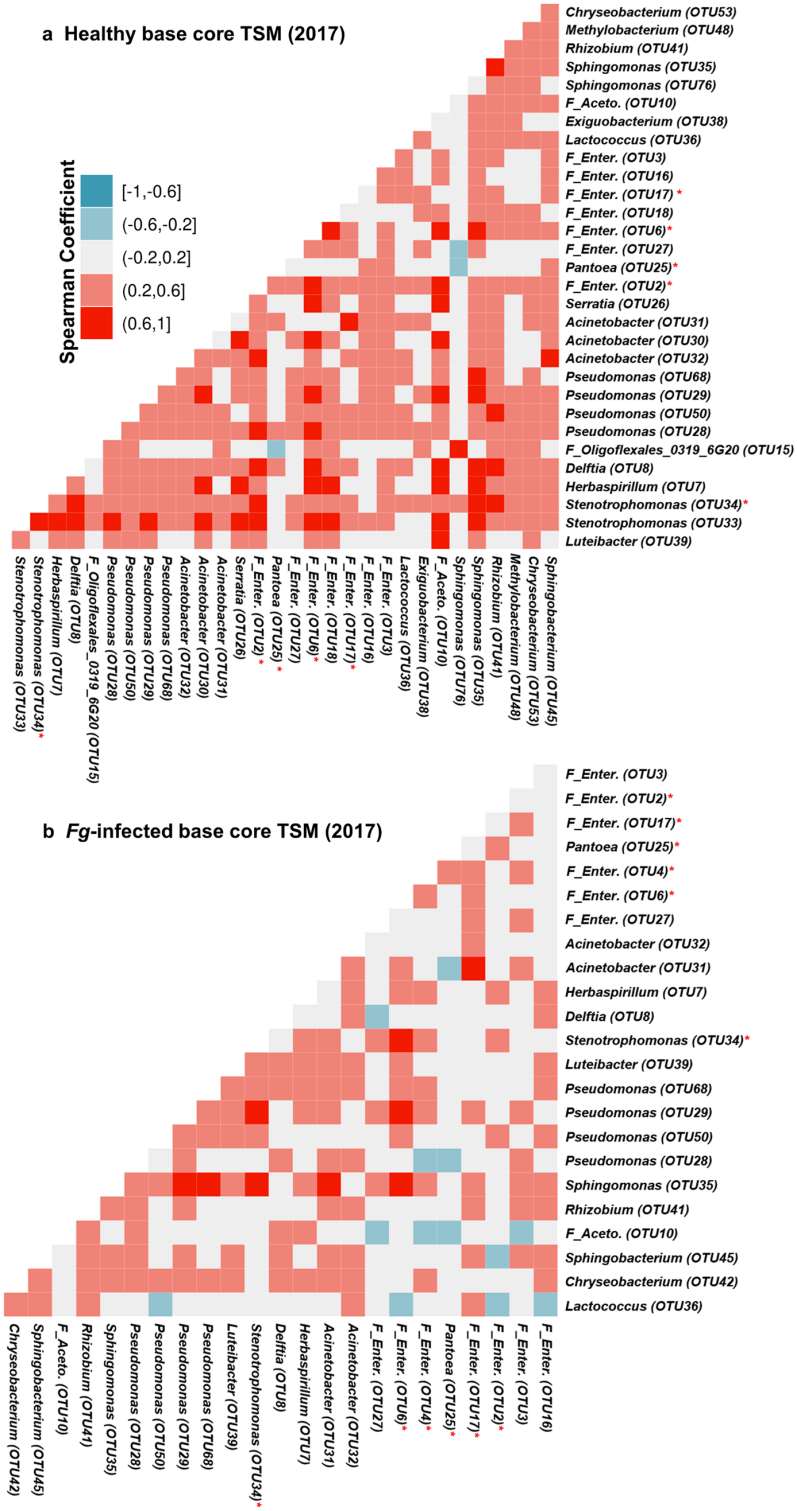
Co-occurrence of core TSM in healthy and *Fg*-infected states. (**a**, **b**) Co-occurrence of core taxa displaying calculated Spearman’s rank correlation coefficients in 2017 for (**a**) healthy base tissues and (**b**) *Fg*-infected base tissues (also see Supplementary Fig. S13a–f).Taxa tagged by a red asterisk denote *Fg*-indicators in silk base, 2017.

To identify candidate microbial networks within the core TSM, Spearman’s rank correlations were used to identify strong co-occurring taxa (coefficient ≥ 0.6 and *p*-value ≤ 0.0001) (Fig. 5a, b; Supplementary Fig. S13). In general, *Fg* infection weakened the co-occurrences between core taxa, especially in the silk base. While the trial year and *Fg* infection affected co-occurring taxa, *Sphingomonas* (OTU35) was consistently the strongest co-occurring taxa. Across tissue samples, OTU35 repeatedly associated with *Pseudomonas* (OTU29, OTU68), suggestive of a consortium, and inconsistently with other core microbiome taxa (see Indicator Bacterial Taxa section below). These results suggest that OTU35 forms the hub of the core TSM.

### Identification of *Fusarium*-indicator bacterial taxa

**Figure 6:**
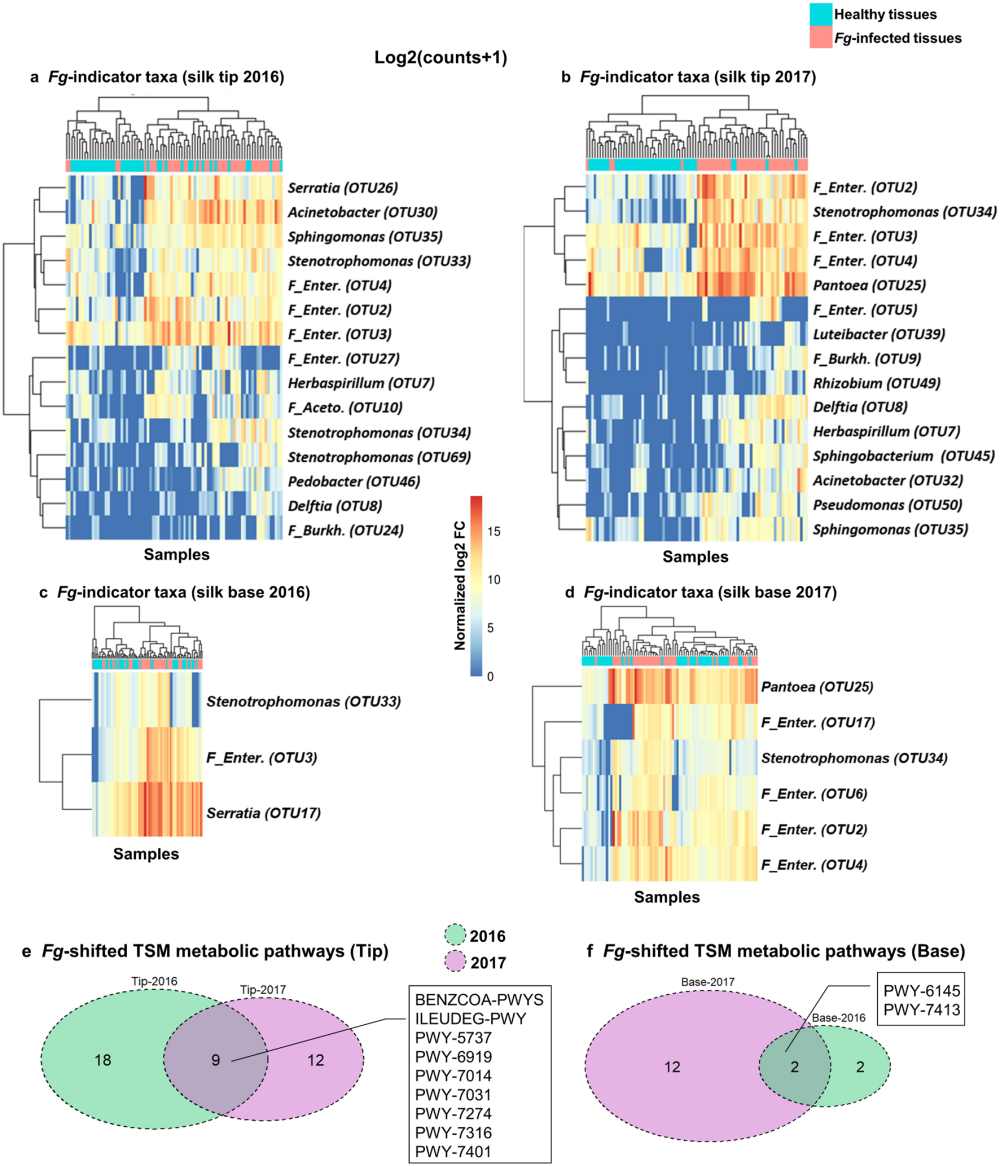
TSM OTUs that significantly change in abundance after exposure to *Fusarium graminearum* infection. (**a**–**d**) Heatmaps of OTUs (*Fg*-indicator taxa) that significantly changed in abundance upon *Fg* infection (Log2 fold change of 16S read counts at *p* value ≥ 0.05) in (**a**) silk tip tissues (2016), (**b**) silk tip tissues (2017), (**c**) silk base tissues (2016), and (**d**) silk base tissues (2017). X-axis represents samples of silk tissues. Impacts of *Fg* infection on TSM predicted metabolic pathways. (**e**, **f**) Venn diagrams displaying year-to-year sharing of predicted TSM metabolic pathways that significantly changed upon *Fg* infection of (**e**) silk tip tissues, and (**f**) silk base tissues (see Supplementary Table S7 for pathway information, and Supplementary Fig. S16 for extended error bars).

Given the overall proliferation of the TSM in response to *Fg*, next we identified the specific taxa that were responsible for this proliferation, hypothesizing that these taxa may protect the host tissue against *Fg* attack. We computed the log2 fold change of taxa abundance using the DESeq2 R package at an adjusted *p*-value < 0.05 (Supplementary Information). Interestingly, the taxa that increased consistently in both 2016 and 2017 in response to *Fg* infection (Supplementary Table S6) were generally members of the core TSM (both healthy and/or diseased state), specifically *Enterobacteriaceae* (OTU2, OTU3, OTU4), *Herbaspirillum* (OTU7), *Delftia* (OTU8), *Stenotrophomonas* (OTU34), and *Sphingomonas* (OTU35) (identified above as the microbiome hub) (Fig. 6a–d)*.* Other taxa that increased in only a single year included consistent core members such as *Pantoea* OTU25 (the most prevalent taxa in the TSM) and *Enterobacteriaceae* (OTU6).

The indicator results were independently confirmed using the q2-sample-classifier, a plugin in a supervised machine learning method (Qiime2 platform) for predicting sample characteristics based on the change in microbiome composition upon *Fg* infection. The top 20 important features/OTUs that distinguish between healthy and *Fg-*infected samples were selectively visualized in heatmaps along with Area Under the Receiver Operating Characteristics (AUROC) graphs to check the performance of the classification model (Supplementary Figs. S14, S15). The predictions showed that tip sample groups had 14 OTUs that overlapped across trial years including the *Fusarium-*indicator taxa calculated above (using the Deseq2 R package) along with other taxa, mostly part of the shared core including *Pseudomonas* (OTU28) and *Lactococcus* (OTU36). For the silk base tissues, only 3 OTUs [*F_Enterobacteriaceae* (OTU4), *Pseudomonas* (OTU29), and *Methylobacterium* (OTU48)] were shared across trial years with a lower discrimination capacity between the healthy and *Fg*-infected tissue samples (Supplementary Fig. S15).

Some of the *Fusarium*-indicator taxa strongly but inconsistently co-occurred with OTU35 (coefficient ≥ 0.6 and    *p*-value  ≤ 0.0001) in *Fg*-infected tissues, suggestive of a silk-protecting consortium of up to 8 taxa as candidate members, including *F_Enterobacteriaceae* (OTU4, OTU17), *Pantoea* (OTU25), and *Delftia* (OTU8) (Fig. 5a, b; Supplementary Fig. S13).

### Shifts in predicted metabolic pathways upon *Fusarium* infection

Taking into account all sample groups, the reduction in TSM taxa upon *Fg* infection was associated with a reduction in 44 bacterial metabolic pathways and an increase in only one pathway (silk base 2016; dTDP-6-deoxy-α-d-allose biosynthesis). Nine pathways were consistent across years in the tip, and two pathways were consistent in the base tissue (Fig. 6e, f, Supplementary Fig. S16, Supplementary Table S7). Specifically, *Fg* infection was predicted to cause the loss of 17 antibiotic biosynthesis pathways along with additional pathways related to macronutrient cycling (carbohydrates, amino acids, phospholipids) (Supplementary Fig. S16, Supplementary Table S7). These predictions were obtained from bioinformatic metabolic pathway analysis (q2-picrust2, STAMP)^24,25^ using 2-sided White’s non-parametric t-test adjusted for 2-group comparisons with a Bonferroni’s correction method.

## Discussion

A healthy style is essential for plant sexual reproduction. Ancient peoples of the Americas selected for elongated corn styles (silks) and understood their importance, depicting them as hairs on deity statues^26^. Today, we estimate that there are > 4000 km of silks per U.S. acre of corn. Here we reported that the transmitting style microbiome (TSM) of maize is dynamic and complex, spanning the prokaryotic tree of life (Supplementary Fig. S4)^23^. The TSM population was observed to host 1,300 bacterial genera, but surprisingly only 7–11 taxa accounted for ~ 45–69% of the entire microbiome based on mean relative abundance (Fig. 4b). Nine of these were amongst the most widespread silk taxa, prevalent across diverse temperate maize genotypes, spatial field blocks, trial year, and fungal attack (Fig. 4c, d). Six of the most abundant taxa belong to one evolutionary branch (*Enterobacteriales)*, including OTU25 (*Pantoea*) which occupied 15–26% of the healthy TSM and was prevalent across 99% of all silk samples (Fig. 4b).

### Taxonomic comparison to previous studies

Taxonomically, the TSM showed some similarities but also significant differences compared to the style microbiomes of other plants. Similar to a recent elegant study involving apple styles^9^, we observed that *Proteobacteria* dominated the TSM, in particular the *Enterobacteriaceae* (Fig. 1, Table 1). However, in apple styles, whereas the only other dominant family was the *Pseudomonadaceae*, here the other dominant and consistent families included not only *Pseudomonadaceae*, but also *Moraxellaceae*, *Burkholderiaceae*, *Sphingomonadaceae*, *Xanthomonadaceae*, *Acetobacteraceae* (Table 1, Supplementary Table S3). Perhaps this difference is explained by the narrow sampling of the apple study (9 trees of one cultivar). In the wild Hawaiian tree, *Metrosideros polymorpha*, the styles and stamens of 33 individual trees were dominated by *Enterobacteriaceae*, *Bradyrhizobiaceae*, *Staphylococcaceae*, *Micrococcaceae*, *Porphyromonadaceae*, *Rhodobacteraceae*, and *Sphingomonadaceae*^7^, showing some overlap but also differences to the maize TSM. In wild monkeyflowers (*Mimulus guttatus*), the stamens and styles were dominated by OTUs from the *Pseudomonadales* order, with core microbes including *Acinetobacter*, *Pseudomonas*, *Bacteroides* and *Corynebacterium*^8^. Cultured epiphytic bacteria from pear styles were dominanted by *Pseudomonas*^6^. Unlike the maize TSM, a hallmark of these previous studies^7,9^ is significant variation among samples of dominant microbes, even from a narrow host genotype^9^, perhaps because the stigma and style tissues sampled were all exposed to the environment unlike this current study.

### Potential functions of the transmitting style microbiome

There may have been strong long-term selection pressure on the TSM to increase host fitness by assisting the style to achieve successful fertilization and to reduce its vulnerability to invading pests and pathogens, well known for maize silks. Interestingly, many of the top 50 dominant TSM genera (based on relative abundance, Supplementary Fig. S6, Table 1, Supplementary Table S3a, b) were previously reported as biocontrol agents^18,27–30^ including those shown to have anti-*Fusarium* activity via diverse biological mechanisms^31,32^ (elaborated below). Specific dominant TSM genera (*Bacillus*, *Paenibacillus*, *Serratia* and *Pseudomonas*) are known to produce volatile compounds (e.g., 2,3-butanediol) that elicit the host immune response^33,34^. Some of the dominant TSM taxa were previously reported to secrete insecticides [e.g., *Bacillus* (mainly *B. thuringiensis*), *Paenibacillus*, *Serratia*, *Pseudomonas*, *Burkholderia*, *Streptomyces*] against a variety of pests including armyworms and corn rootworms^35^. In terms of abiotic stress, silk growth is impeded by low nitrogen availability, perhaps explaining the surprising presence of many well-known nitrogen-fixing genera including *Rhizobium*, *Bradyrhizobium, Herbaspirillum, Azospirillum*, *Methylobacterium, Bacillus*, *Paenibacillus*, *Brevundimonas, Massilia, Achromobacter, and Novosphingobium*^36^. Similarly, maize silk growth is highly susceptible to drought, and the top 50 genera in silks include those previously shown to increase in abundance under drought in the roots of 19 grasses including maize (e.g. *Stenotrophomonas*, *Serratia*, *Achromobacter*, *Sphingobacterium*, *Streptomyces*, *Staphylococcus*, *Paenibacillus,* and *Bacillus*)^37^. These and other core genera (*Pantoea*, *Sphingomonas*, *Burkholderia, Enterobacter*, *Azospirillum*) have been shown to promote drought tolerance in plants^38–40^. For example, mechanistically, *Azospirillum* has been shown to trigger abscisic acid production^38^, while *Rhizobium* upregulates trehalose-6-phosphate synthase^39^. It is also interesting to speculate whether the dominant genera contribute to the extraordinarily rapid growth of silks, analogous to root-stimulating rhizobacteria, as several of these (*Pseudomonas, Serratia, Stenotrophomonas, Bacillus, Paenibacillus, Rhizobium, Sphingomonas*) synthesize growth phytohormones such as indole-3-acetic acid (IAA), cytokinin, and/or gibberellins^41^. Other core taxa may be regulating the silk response to abiotic/biotic stress by modulating ethylene levels^41^. Perhaps some of the well-known silk medicinal and phytochemical compounds used by traditional societies including in silk teas^13,42^ are actually associated with the silk microbiome, alone or interacting with the host, to serve some of the host-protective or ecological functions noted above. Finally, it will be interesting to test whether members of the TSM assist or interfere in reproduction directly, perhaps as a vestige of early plant evolution. For example, some silk taxa produce compounds known to be required for pollen tube attraction to the embryo sac, including gamma-aminobutyric acid (GABA) (*Lactobacillus*, *Lactococcus*)^43^, or they may secrete enzymes implicated in pollen grain penetration into the silk (e.g. xylanase by *Pantoea*)^44^, or polypeptides with β-expansin activity needed for stigma penetration and pollen tube growth to the egg sac (e.g., *Bacillu*s, *Streptococcus*, *Streptomyces*, *Staphylococcus*, *Enterococcus*, *Lactobacillus*, *Lactococcus*, *Clostridium*, *E. coli*, *Pseudomonas* and *Flavobacterium*)^45^.

### Interaction between the TSM and *Fusarium* pathogen

*Fusarium graminearum* silk infection resulted in a dramatic reduction in TSM diversity (Table 1, Supplementary Table S3a, b) but also a doubling in total 16S read counts (Supplementary Table S2). This is contradictory to apples, where exposure of stigma/style tissue to the pathogen *Erwinia amylovora* resulted in a shift in the microbiome composition, but no significant effect on microbiome community diversity compared to the water control^9^. There are several possible reasons for the diversity loss observed here. First, *Fg* may be competing for specific nutrients. Second, *Fg*-derived mycotoxins/antibiotics may be killing specific classes of bacteria or disrupting bacterial cell–cell communication (Quorum Sensing) responsible for networking^46,47^. Indeed, upon *Fg* infection, there was an observable reduction in the co-occurrence of core TSM members (i.e. disruption of the core network) (Fig. 5, Supplementary Fig. S13). Third, silk cells may be emitting damaging reactive oxygen species during the host defence response which some bacterial taxa are sensitive to. Finally, perhaps the rare taxa provide a diverse genetic repertoire to improve host fitness under different environmental stresses^48^, but upon artificial *Fusarium* infection only a core protective microbiome is amplified (Fig. 6a–d, Supplementary Fig. S15). In fact, 7 taxa were consistently and significantly responsible for the increased abundance upon infection (indicator taxa), including members of the core microbiomes (Fig. 4c, d). We previously showed that an *Enterobacter* sp. could suppress *Fg* when sprayed onto silk tips^20^, consistent with the finding here that *Enterobacteriaceae* species (OTU2, OTU3, OTU4) consistently increased in abundance upon *Fg* attack in silk tips^20^. Similarly, several of these taxa have previously been reported to suppress *Fusarium* species including: *Pseudomonas* (OTU50)^30^; *Pantoea* (OTU25, the most prevalent and abundant TSM taxa, which significantly increased in abundance in 2017) that was previously shown to exhibit anti-*Fusarium* activity through a variety of mechanisms including chitinolytic activity and regulation of *Fusarium* virulence/cell division genes^32, 49–51^; and *Delftia* (OTU8) that has previously been reported to antagonize diverse fungal pathogens^52^. In apple, where abundance of an *Erwinia* pathogen was carefully quantified, no strong correlation was observed between individual silk/style microbiome OTUs and *Erwinia* titre, leading them to hypothesize that the microbiome regulated pathogen activity rather than its abundance, though the study suffered from significant sample variation^9^.

It is noteworthy that of the *Fg*-responsive taxa, *Sphingomonas, Pseudomonas* sp., *Acinetobacter* sp.*, Delftia,* and *Stenotrophomonas* also naturally dominate the human female reproductive tract^1^ which serves an analogous reproductive function as silks and are similarly invaded by fungal pathogens. Interestingly, *Sphingomonas* (OTU35) consistently co-occurred across infected and healthy samples, with multiple taxa indicating it as a microbiome-hub in a consortium with *Pseudomonas, Acinetobacter* and *Stenotrophomonas* (as well as *Pantoea*, *Rhizobium, Pedobacter*, and other members of the *Enterobacteriaceae* family) (Fig. 5a, b; Supplementary Fig. S13). All these co-occurring taxa were previously reported individually to antagonize *Fusarium*^53–59^. Of note, various anti-*Fg* strains have additive or synergistic effects, as suggested by previous studies^60^. Others function by biodegrading and detoxifying *Fusarium* mycotoxins: for example, S*phingomonas* and fluorescent Pseudomonads decrease deoxynivalenol (DON) mycotoxin^58,61^, while a *Lactococcus* sp. has been shown to detoxify zearalenone (ZEA) mycotoxin in cereal crops^62^. Alternatively, some of the *Fg*-indicator taxa may be inducing host defence, work as part of a consortium, or otherwise directly protect the silk host tissue.

Metabolic pathway predictions suggested that *Fg* infection resulted in significant loss of TSM functionality, including antibiotic biosynthesis pathways and nutrient recycling pathways (Supplementary Fig. S16; Supplementary Table S7). It may be that *Fg*-induced dysbiosis depletes the genetic repertoire encoded by the TSM, especially activities not beneficial during fungal pathogen attack (e.g. antibiotic synthesis)^48, 63^. This result is consistent with the dramatic loss in rare TSM taxa following *Fg* inoculation.

One limitation of this study was that Agral surfactant was added to the *Fusarium* treatment but not the control treatment, to help *Fusarium* spores attach to the silks; we cannot rule out Agral also affected the TSM. However, the *Fusarium* formulation was applied only to the exposed silks, whereas the collected silk tissues were distal (husk leaf-protected), so, the sampled tissues used for the microbiome study were not directly exposed to Agral. Furthermore, all plants were watered every 8–10 min through installed misting systems during the entire experiment, and thus the Agral was likely washed out immediately.

### Year to year variation in the TSM

The anthesis silk interval (ASI)—the difference in timing between silk emergence and pollen shed (synchronization)—critically impacts the success of fertilization and hence grain yield in corn^11^. Silk emergence is limited by environmental stress including water and nitrogen limitation^64^. In addition, climatic variability impacts the ASI, potentially causing dramatic reductions in yield^64^. Here, the recorded fluctuation in climatic conditions across the two years of field trials (Supplementary Figs. S1, S2) was associated with a significant year-to-year shift in the TSM of the silk tip, most likely attributable to shifts of the non-overlapping taxa (Fig. 3a–d; Supplementary Fig. S7a–l, Supplementary Table S5a). These results suggest that the external environment can impact the TSM (Supplementary Table S5a). The impact of seasonality on the stigma/style microbiome was not examined in previous studies in other plants^7–9^, however in apples, it was shown that time after petal opening (and hence presumably environmental exposure) reduced stigma microbiome diversity^9^. In the current study, there were year-to-year differences in candidate anti-*Fg* taxa that increased in abundance upon *Fg*-infection (Fig. 6a–d, Supplementary Table S7). *Fg* disease severity (gibberella ear rot) and DON mycotoxin levels in corn are well known to vary annually, and it has been very difficult to develop predictive models^65^. Human/livestock health episodes have been associated with *Fg*-derived mycotoxins^66^. This is the first study to suggest that year-to-year fluctuation of the TSM may be a contributing factor to this global threat to human/livestock health, food security and farmer livelihoods. Testing this hypothesis is particularly urgent given climate change.

### Summary and future perspectives

In plant reproduction, healthy style tissue at the pollen nuclei transmission stage is critical for successful fertilization^5^. The style microbiome in the world’s most important cereal crops was not previously reported. Given its inherent advantages compared to many other plants, this study establishes the maize silk as a model for fundamental and applied research of plant reproductive microbiomes including from cereals. The study demonstrates that healthy transmitting maize silks possess thousands of bacterial taxa, which we termed the transmitting silk microbiome (TSM). The TSM is dominated by a small but abundant core microbiome that is prevalent across diverse host genotypes (heterotic groups) in modern maize. Maize silks at the transmission stage are susceptible to pathogen invasion including from *Fusarium graminearum* (*Fg*). Despite huge investments made over decades to combat *Fg* in maize globally, it remains a billion-dollar problem during outbreak years. Here, *Fg* infection was associated with a dramatic decrease in TSM microbial diversity and perturbed predicted metabolic functionality, but surprisingly a doubling of total TSM read counts, primarily by elevating much of the core microbiome (7–25 MiSeq-ASVs), suggestive of a selective microbiome response against *Fusarium*. Since the core microbiome dominated read counts, and the rarer taxa varied significantly across samples (sample dispersion), statistically, *Fg* invasion was not associated with a significant change in TSM community composition, but this masked the actual drama in the microbiome. Future studies are needed to understand whether the elevated TSM taxa are protecting themselves, their host tissues, and/or directly combating *Fusarium* and its mycotoxins. Given that *Fg* associated disease severity is known to vary year to year, it is noteworthy that the TSM in silk tips showed significant yearly variation under field conditions. If it is protective, the seasonally dynamic silk microbiome may be part of the problem and solution.

Moving forward, the identification of the healthy TSM and/or a consortium of candidate anti-*Fg* taxa should be considered as targets for direct selection in maize breeding programs using taxa-specific molecular markers—a novel approach to disease resistance breeding in crops. Such a strategy could also be explored in SubSaharan Africa where silk invading fungal pathogens such as *Fusarium* and *Aspergillus* are major sources of human carcinogens^67^. In temperate regions, DON mycotoxin has been shown to disrupt human and livestock gut microbiomes, in part explaining its gastro-intestinal impacts and its nickname vomitoxin^4^. Given the results of this study, it is interesting to hypothesize whether the DON antibiosis effect in humans/animals is the indirect byproduct of *Fg* co-evolving in an arms race with the defensive microbiome of its native plant habitat (e.g. silks). Finally, given the silks in this experiment were open-pollinated, it will be interesting to explore the relative maternal and paternal contributions to the TSM. These and many other fundamental and applied questions now arise that should be explored about the functionality and origin of the TSM as well as the style microbiomes of other plants.

## Methods

### Field experimental design

A total of 14 corn genotypes were included in this study: eleven were Agriculture and Agri-Food Canada (AAFC) maize inbred lines, provided by Dr. Lana Reid along with 3 commercial hybrids to add relevance to the study (Supplementary Table S1). These genotypes represent a diversity of heterotic groups in modern maize and have a diversity of resistance to *F. graminearum* (*Fg*, Supplementary Table S1)*.* Seeds were grown in the summer of 2016 and 2017 in a completely randomized, split-block design with three replicate blocks, at Ridgetown Station, University of Guelph, Canada. Each open-pollinated block consisted of 2 rows with guard row(s) surrounding all the blocks. Each row had all the genotypes (each 2 m long, with 20 seeds, followed by a small space); in the adjacent paired row, the genotypes were randomized, and hence each genotype had duplicate plots. One of the plots was treated with *Fg*, while the other was the control. No one row was dedicated to the *Fg* or control treatments; rather they were randomized across the paired rows within each block, and randomized again between blocks. For each genotype and for each of the 3 blocks, per plot, up to 16 plants (minus the outer 2 plants at each edge) were treated with *Fg,* while 16 in the adjacent row acted as the control (not sprayed). Pure *Fusarium* inoculum was applied after silk emergence when the silks of the primary ear presented the first visual signs of senescence (faint brown color) (Supplementary Figs. S1, S2). Approximately 2 ml of *Fg* inoculum (20,000 spores/ml) was sprayed directly onto the silks from a bottle held at roughly a 3 cm distance. The inoculation date varied between blocks based on this silk maturity criterion, but ranged from August 15–18 in 2016, and August 11–14 in 2017. Each cob was inoculated with *Fg* twice over 24–48 h. Plots were irrigated using overhead misting prior to and after inoculation until harvest (an automated cycle of 30 s on, and 8–10 min off, from 10:00 to 18:00 h). The system was operated at 206 kPa, and the nozzles delivered approximately 0.6 L/min of water. Harvesting occurred on August 25 in 2016, and August 21 in 2017. For silk sampling, 3 cobs from each plot (3 separate plants) were chosen randomly that were also representative of the average cob size for that plot. In the lab, under sterile conditions, silks from all 3 cobs per plot were pooled and frozen at − 80 °C for later DNA isolation (see Tissue harvesting and DNA isolation, below). The weather information was obtained from the official website of Government of Canada, Environment and Natural Resources (https://weather.gc.ca/) (Supplementary Figs. S1, S2).

### *Fusarium* spore isolation and inoculum preparation

Three local *Fg* isolates were used to prepare pure inoculum under in vitro sterile conditions: one isolate from infected corn and two from wheat from Southern Ontario, Canada . All three isolates produced clear disease symptoms in their respective hosts during the previous growing season. The *Fg* strains were purified via serial subculturing on PDA agar plates. First, the seeds were surface-sterilized, then plated for several days. The leading edge of the fungal growth was picked for culturing on fresh PDA agar plates. The spores were then harvested, and diluted until single spores could be resolved on water agar. For further purification, single spores were picked and plated individually on fresh PDA agar plates as a pure culture per spore. To confirm the purity of spores prior to the inoculum preparation, they were microscopically identified, then small cubes were aseptically cut from the plates and used to inoculate flasks of autoclaved spore broth (modified Bilay’s medium)^68^ then incubated for 3–5 days at 25 °C under shaking. The fungal spores from each flask were visualized under the microscope and counted using a hemocytometer to check the purity and to adjust the spore suspension concentration. To prepare the final cocktail of *Fg* spores, each of the three adjusted spore suspensions was combined equally to produce a final suspension concentration of 20,000 spores/ml, then 1.0% v/v Agral ® 90 (Syngenta Canada Inc.) was added as a surfactant.

### Tissue harvesting and DNA isolation

For every single replicate, three ears were harvested from each subplot in the summer season (Supplementary Fig. S1). The exposed silk tissues were removed from each cob and the length of each ear was measured to be cut into three equal portions (Fig. 1c). Then the silk tip (front portion) tissues were pooled separately from the silk base tissues (back portion), and each was consolidated in a single Petri dish and marked with a unique sample ID. All samples were stored at − 80 °C for DNA isolation. Qiagen DNeasy Plant Mini Kit was used for DNA extraction from silk tips (100 mg of tissue according to the kit protocol), whereas a CTAB protocol (see Supplementary Information) was used for silk base tissues (300 mg), because the yield from the Qiagen kit from base tissues was insufficient for high throughput 16S rRNA sequencing^69^. Then, DNA samples were quantified using a Qubit v1.2 fluorometer.

### 16S rRNA amplicon sequencing using MiSeq technology and sequences analysis

DNA samples were submitted to Metagenom Bio Inc. (Waterloo, Canada) for high throughput 16S amplicon sequencing using the Illumina MiSeq platform. Hypervariable region V4 of 16S rRNA genes were amplified using the barcoded primer sets, 515FB: GTGYCAGCMGCCGCGGTAA, 806RB: GGACTACNVGGGTWTCTAAT^70^. PCR was conducted in triplicate for each sample (25 μl each). Each reaction mixture contained 2.5 μl of 10 × standard Taq buffer (New England Biolabs), 0.5 μl of 10 mM dNTP, 5 μl of 1 μM forward primer, 5 μl of 1 μM reverse primer, 1 μl of 25 μM pPNA, 1 μl of 25 μM mPNA^71^ (PNA Bio Inc, Newbury Park, CA), 2 μl DNA, 0.2 μl of BSA (20 mg/ml), 0.2 μl of Taq DNA polymerase (5 units/μl, NEB) and 7.6 μl of PCR water. DNA was denatured at 95 °C for 5 min, followed by 35 cycles of 95 °C for 30 s, 50 °C for 30 s and 72 °C for 50 s, and then extended at 72 °C for 10 min. Each PCR product representing a correct-size amplicon was gel purified on 2% TAE agarose, quantified using the Qubit dsDNA HS Assay Kit (Thermo Fisher Scientific Inc.), then the 3 technical replicates were pooled in equimolar amounts. Then, the library DNA was sequenced with a MiSeq Reagent Kit v2 (2 × 250 cycles) and FASTQ files were generated for taxonomic analysis.

### Bioinformatic analysis

Generated MiSeq raw reads were assigned to their original samples and quality-controlled using Quantitative Insights Into Microbial Ecology Qiime2 core-2019.10 (https://docs.qiime2.org/2019.10/)^72^ with plugins demux (https://github.com/qiime2/q2-demux) for demultiplexing and DADA2^73^ to denoise and dereplicate paired-end sequences. Given the use of 2 different DNA extraction protocols, sequences were filtered at different quality scores; QS30 for silk tip sequences and QS25 for silk base sequences. To generate taxonomy tables, the taxonomic assignment was performed using a Naive Bayesian Classifier trained against SILVA-v132 (https://www.arb-silva.de/documentation/release-132/). Representative sequences were aligned and phylogenetic trees were generated using plugins alignment (MAFFT program)^74^ and phylogeny (FastTree)^75^, respectively. Data files were exported in QIIME2 and consolidated into a standard format (biom-format)^76^, then uploaded as a phyloseq object in R for further microbiome analysis and visualization.

Phyloseq (v.1.22.3), metacoder (0.2.1.9005), vegan (v.2.5-2), ggplot2 (v.3.0), and VennDiagram R packages^77–81^ were used for exploring the maize silk microbiome, and visualizing the core microbiome identified at a 50% prevalence threshold. Diversity analyses (alpha and beta diversity indices) were calculated in Qiime2 platform using the qiime diversity core-metrics-phylogenetic plugin. Principle coordinate analysis (PCoA) was applied on Bray–Curtis and UniFrac distance matrices to visualize the impact of seasonality along with *Fg*-infection on the dynamics of the transmitting silk microbiome.

### Identification of indicator taxa

Taxa that significantly increased in their read counts upon *Fg* treatment were estimated along with the corresponding log2 fold change (log2 FC) in their abundance and adjusted *p*-values (significance level, *p* < 0.05) for multiple testing using the DESeq2 (v.1.18.1) R package^82^. Taxa with less than a total of 10 counts were excluded from the analysis. Estimations were computed for each tissue silk location in each year, separately. The default multiple inference correction, the Benjamini–Hochberg method, was used; the false discovery rate (FDR) was set to 0.015, and heatmaps of log2 (count + 1) of estimated indicator taxa were constructed and visualized as in Fig. 6a–d. Independently, to confirm the results, the supervised machine learning method in the Qiime2-2019.10 pipeline was used to estimate the top 20 significantly discriminative features between healthy and *Fg*-infected samples (the most important features of the silk microbiome that changed significantly in abundance upon treatment with *Fusarium* spores). Generated heatmaps of the top 20 discriminative features (OTUs) were displayed (Supplementary Fig. S15).

### Correlation patterns of either healthy or *Fg*-infected core microbiome

Co-occurrence patterns of the identified core microbiome at a 50% prevalence threshold for each silk tissue location according to fungal pathogen treatment were estimated using the microbiome R package (v.1.0.2)^83^, based on Spearman’s rank correlation with an adjusted *p* value threshold of < 0.05. The computed correlation coefficients were visualized using the GGally R package (v.1.4.0)^84^ as illustrated in Fig. 5, and Supplementary Fig. S13.

### Functional inferences of 16S rRNA gene sequences

For metabolic profile prediction of the TSM upon *Fg* treatment, the q2-picrust2 plugin^24^ was used through the Qiime2-2019.10 pipeline. PICRUSt2 is a bioinformatic tool used for metagenome inferences from the taxonomic profile of a microbial community to predict its functional composition. The algorithm first aligns amplicon sequence variant (ASV) sequences with the reference 16S rRNA gene sequences using HMMER, then the aligned sequences are placed into a reference phylogenetic tree (generated from reference 16S sequences from the Integrated Microbial Genomes (IMG) database). This phylogenetic tree is used to predict genomes and infer traits for each unknown ASV. In the q2-picrust2 plugin, the output comprises three Qiime2 artifacts: ec_metagenome.qza (predictions of enzyme commission metagenome), ko_metagenome.qza (predictions of KEGG orthology), and pathway_abundance.qza (predictions of MetaCyc pathway abundance)^85^. Generated pathways and their corresponding values (copy numbers) for healthy and diseased tissues of each sample group across 2016 and 2017 (tip and base) were analyzed using Statistical Analysis of Metagenomic Profiles (STAMP) software^25^. Analysis was performed using: two-group analysis (healthy versus *Fg*-infected samples), two-sided, White’s non-parametric t-test (statistical hypothesis test), Bonferroni (multiple test correction method), DP: bootstrap (confidence interval method) along with a q-value filter > 0.05, effect size filter (ratio of proportions) < 1, and 95% confidence interval. Extended error bar plots were used to indicate significantly affected metabolic pathways. Overlaps across identified predicted metabolic pathways in both 2016 and 2017 for each silk tissue location (tip and base) were visualized using a Venn-diagram created using the VennDiagram R package. All calculated metabolic pathways were listed in Supplementary Table S7, including their detailed descriptions.

### Statistical analysis

For the mean relative abundance calculations, they were estimated by calculating the relative abundance of a taxon at a specific taxonomic level in each sample, then by calculating the mean relative abundance of that taxon across samples within the same group (Figs. 1d, 4b, Supplementary Fig. S9). For estimating taxa at each taxonomic level (5 levels from the phylum to genus level) that significantly changed in abundance upon *Fg* infection, feature tables with taxonomy of each sample group were collapsed in Qiime2-2019.10 using the qiime taxa collapse function, then uploaded along with their corresponding metadata files in STAMP for statistical analyses using a two-group analysis (healthy versus *Fg* infected samples), two-sided, White’s non-parametric t-test (statistical hypothesis test), Benjamini-Hochberg (multiple test correction method), DP: bootstrap (confidence interval method) along with a q-value filter > 0.05, effect size filter (difference between proportions) < 1, and 95% confidence interval. Extended error bar plots (Supplementary Fig. S10) were used to display the taxa that significantly changed upon *Fg* infection. For alpha diversity analyses, estimation of richness (observed OTUs), diversity (Shannon), evenness (Pielou), and Faith’s Phylogenetic Diversity (FPD) metrics/indices were performed on OTU-tables, and Wilcoxon/Kruskal–Wallis tests were used at *p* value < 0.05. Estimates are available in Supplementary Table S4. For beta diversity analyses, data were normalized using a rarefying method (even sampling depth) for each tested group as illustrated in Supplementary Table S5 to estimate the variation in TSM composition across groups of samples. In the Qiime2-2019.10 platform, a permutational multivariate analysis of variance (PERMANOVA) test was applied to Bray–Curtis, unweighted UniFrac and weighted UniFrac distance matrices with 999 permutations, and the results were confirmed by testing for homogeneity of dispersions through a PERMDISP test. For statistical significance testing, *p* values of < 0.05 were considered significant. To visualize how similar or dissimilar the samples (healthy vs *Fg*-infected) were, 2D Principal Coordinate (PCoA) plots were generated from the calculated matrices. Additional ordination plots were constructed in 5 parallel principal coordinates (vertical lines) to capture the differences between years/treatments where each colored line represents one sample.

### Experimental research including collection of plant material

The authors declare that the collection of plant material used in this study complies with all relevant institutional, national and international guidelines and treaties. The seed germplasm are Canadian domesticated *Zea mays* (modern corn) which is not endangered, and the seed material was bred in Canada by one of the co-authors (Dr. Lana Reid from Agriculture and Agrifood Canada) within the same domestic jurisdiction and hence not in violation of any international treaties for the protection of plant germplasm.

### Statement of permissions and/or licenses for collection of plant or seed specimens

The authors declare that the seed specimens used in this study were generated by one of the study co-authors (Dr. Lana Reid from Agriculture and Agrifood Canada). They are publicly accessible seed materials and we were given explicit written permission to use them for this research. They are part of Dr. Reid’s public corn breeding program. They were not collected from the wild, and instead bred in Canada at Agriculture and Agrifood Canada, a public government institution.

## Supplementary Information

**Supplementary Information**.

**Supplementary Dataset S1**.

## References

[1] Chen C (2017). The microbiota continuum along the female reproductive tract and its relation to uterine-related diseases. Nat. Commun..

[2] Thompson MEH, Raizada MN (2018). Fungal pathogens of maize gaining free passage along the silk road. Pathogens.

[3] Anahtar MN, Gootenberg DB, Mitchell CM, Kwon DS (2018). Cervicovaginal microbiota and reproductive health: the virtue of simplicity. Cell Host Microbe.

[4] Liao Y (2018). Deoxynivalenol, gut microbiota and immunotoxicity: a potential approach?. Food Chem. Toxicol..

[5] Zhou LZ, Juranić M, Dresselhaus T (2017). Germline development and fertilization mechanisms in maize. Mol. Plant.

[6] Stockwell VO (1999). Epiphytic colonization of pear stigmas and hypanthia by bacteria during primary bloom. Phytopathology.

[7] Junker RR, Keller A (2015). Microhabitat heterogeneity across leaves and flower organs promotes bacterial diversity. FEMS Microbiol. Ecol..

[8] Rebolleda Gómez M, Ashman TL (2019). Floral organs act as environmental filters and interact with pollinators to structure the yellow monkeyflower (*Mimulus guttatus*) floral microbiome. Mol. Ecol..

[9] Cui Z, Huntley RB, Zeng Q, Steven B (2020). Temporal and spatial dynamics in the apple flower microbiome in the presence of the phytopathogen *Erwinia amylovora*. ISME J..

[10] Turc O, Bouteillé M, Fuad-Hassan A, Welcker C, Tardieu F (2016). The growth of vegetative and reproductive structures (leaves and silks) respond similarly to hydraulic cues in maize. New Phytol..

[11] Borrás L, Vitantonio-Mazzini LN (2018). Maize reproductive development and kernel set under limited plant growth environments. J. Exp. Bot..

[12] Debruin JL, Hemphill B, Schussler JR (2018). Silk development and kernel set in maize as related to nitrogen stress. Crop Sci..

[13] Rahman NA, Wan Rosli WI (2014). Nutritional compositions and antioxidative capacity of the silk obtained from immature and mature corn. J. King Saud Univ. Sci..

[14] Johnson ET, Berhow MA, Dowd PF (2007). Expression of a maize Myb transcription factor driven by a putative silk-specific promoter significantly enhances resistance to *Helicoverpa zea* in transgenic maize. J. Agric. Food Chem..

[15] Wilson D (2019). Candida albicans. Trends Microbiol..

[16] Patriarca A, Fernández Pinto V (2017). Prevalence of mycotoxins in foods and decontamination. Curr. Opin. Food Sci..

[17] Magan N, Medina A (2016). Integrating gene expression, ecology and mycotoxin production by *Fusarium* and *Aspergillus* species in relation to interacting environmental factors. World Mycotoxin J..

[18] Mousa WK, Shearer CR, Limay-Rios V, Zhou T, Raizada MN (2015). Bacterial endophytes from wild maize suppress *Fusarium graminearum* in modern maize and inhibit mycotoxin accumulation. Front. Plant Sci..

[19] Mousa WK (2015). An endophytic fungus isolated from finger millet (*Eleusine coracana*) produces anti-fungal natural products. Front. Microbiol..

[20] Mousa WK (2016). Root-hair endophyte stacking in finger millet creates a physicochemical barrier to trap the fungal pathogen *Fusarium graminearum*. Nat. Microbiol..

[21] Mousa WK, Schwan AL, Raizada MN (2016). Characterization of antifungal natural products isolated from endophytic fungi of finger millet (*Eleusine coracana*). Molecules.

[22] Chen Y (2018). Wheat microbiome bacteria can reduce virulence of a plant pathogenic fungus by altering histone acetylation. Nat. Commun..

[23] Hug LA (2016). A new view of the tree of life. Nat. Microbiol..

[24] Langille MGI (2013). Predictive functional profiling of microbial communities using 16S rRNA marker gene sequences. Nat. Biotechnol..

[25] Parks DH, Tyson GW, Hugenholtz P, Beiko RG (2014). STAMP: statistical analysis of taxonomic and functional profiles. Bioinformatics.

[26] Taube K (1985). The Classic Maya maize god: a reappraisal. Fifth Palenque Round Table.

[27] Schmidt TM, Thomé AHE, Sperotto RA, Granada CE (2018). Effect of rhizobia inoculation on the development of soil-borne pathogens infecting common bean plants. Eur. J. Plant Pathol..

[28] Shi C (2014). Biocontrol of *Fusarium graminearum* growth and deoxynivalenol production in wheat kernels with bacterial antagonists. Int. J. Environ. Res. Public Health.

[29] Zhao Y (2014). Antagonistic action of *Bacillus subtilis* strain SG6 on *Fusarium graminearum*. PLoS ONE.

[30] Hu W, Gao Q, Hamada MS, Dawood DH (2014). Potential of *Pseudomonas chlororaphis* subsp aurantiaca Strain Pcho10 as a biocontrol agent against *Fusarium graminearum*. Phytopathology.

[31] Lisboa BB (2015). Soil fungistasis against *Fusarium graminearum* under different crop management systems. Rev. Bras. Ciência do Solo.

[32] Díaz Herrera S, Grossi C, Zawoznik M, Groppa MD (2016). Wheat seeds harbour bacterial endophytes with potential as plant growth promoters and biocontrol agents of *Fusarium graminearum*. Microbiol. Res..

[33] Chung J, Song GC, Ryu C-M (2016). Sweet scents from good bacteria: Case studies on bacterial volatile compounds for plant growth and immunity. Plant Mol. Biol..

[34] Cho SM (2008). 22R,3R-butanediol, a bacterial volatile produced by *Pseudomonas chlororaphis* O6, is involved in induction of systemic tolerance to drought in *Arabidopsis thaliana*. Mol. Plant-Microbe Interact..

[35] Ruiu L (2018). Microbial biopesticides in agroecosystems. Agronomy.

[36] Rosenblueth M (2018). Nitrogen fixation in cereals. Front. Microbiol..

[37] Naylor D, Degraaf S, Purdom E, Coleman-Derr D (2017). Drought and host selection influence bacterial community dynamics in the grass root microbiome. ISME J..

[38] Cohen AC (2015). *Azospirillum brasilense* ameliorates the response of *Arabidopsis thaliana* to drought mainly via enhancement of ABA levels. Physiol. Plant..

[39] Suárez R (2008). Improvement of drought tolerance and grain yield in common bean by overexpressing trehalose-6-phosphate synthase in Rhizobia. Mol. Plant-Microbe Interact..

[40] Rho H (2018). Do endophytes promote growth of host plants under stress? A meta-analysis on plant stress mitigation by endophytes. Microb. Ecol..

[41] Santoyo G, Moreno-Hagelsieb G, del Carmen Orozco-Mosqueda M, Glick BR (2016). Plant growth-promoting bacterial endophytes. Microbiol. Res..

[42] Chagas FO, Pessotti RDC, Caraballo-Rodríguez AM, Pupo MT (2018). Chemical signaling involved in plant-microbe interactions. Chem. Soc. Rev..

[43] Dhakal R, Bajpai VK, Baek KH (2012). Production of GABA (γ-aminobutyric acid) by microorganisms: a review. Brazilian J. Microbiol..

[44] Ma J (2016). Genomic and secretomic insight into lignocellulolytic system of an endophytic bacterium *Pantoea ananatis* Sd-1. Biotechnol. Biofuels.

[45] Georgelis N, Nikolaidis N, Cosgrove DJ (2015). Bacterial expansins and related proteins from the world of microbes. Appl. Microbiol. Biotechnol..

[46] Liew W, Mohd-redzwan S (2018). Mycotoxin: its impact on gut health and microbiota. Front. Cell. Infect. Microbiol..

[47] Bacon CW, Hinton DM, Mitchell TR (2017). Is quorum signaling by mycotoxins a new risk-mitigating strategy for bacterial biocontrol of *Fusarium verticillioides* and other endophytic fungal species?. J. Agric. Food Chem..

[48] Lynch MDJ, Neufeld JD (2015). Ecology and exploration of the rare biosphere. Nat. Rev. Microbiol..

[49] Pandolfi V, Jorge EC, Melo CMR, Albuquerque ACS, Carrer H (2010). Gene expression profile of the plant pathogen *Fusarium graminearum* under the antagonistic effect of *Pantoea agglomerans*. Genet. Mol. Res..

[50] Gohel V, Singh A, Vimal M, Ashwini P (2006). Bioprospecting and antifungal potential of chitinolytic microorganisms. Afr. J. Biotechnol..

[51] Walterson AM, Stavrinides J (2015). *Pantoea*: Insights into a highly versatile and diverse genus within the Enterobacteriaceae. FEMS Microbiol. Rev..

[52] Han J (2020). Characterization of a novel plant growth-promoting bacteria strain *Delftia tsuruhatensis* HR4 both as a diazotroph and a potential biocontrol agent against various plant pathogens. Syst. Appl. Microbiol..

[53] Siegel-Hertz K (2018). Comparative microbiome analysis of a Fusarium wilt suppressive soil and a Fusarium wilt conducive soil from the Châteaurenard region. Front. Microbiol..

[54] Adame-García J, Luna-Rodríguez M, Iglesias-Andreu LG (2016). Vanilla rhizobacteria as antagonists against *Fusarium oxysporum* f. sp. vanillae. Int. J. Agric. Biol..

[55] Verma SK (2018). Bacterial endophytes from rice cut grass (*Leersia oryzoides* L.) increase growth, promote root gravitropic response, stimulate root hair formation, and protect rice seedlings from disease. Plant Soil.

[56] Devi AR, Sharma GD, Majumdar PB, Pandey P (2018). A multispecies consortium of bacteria having plant growth promotion and antifungal activities, for the management of Fusarium wilt complex disease in potato (*Solanum tuberosum* L.). Biocatal. Agric. Biotechnol..

[57] Adeniji AA, Babalola OO (2018). Tackling maize fusariosis: in search of *Fusarium graminearum* biosuppressors. Arch. Microbiol..

[58] He WJ (2017). An aldo-keto reductase is responsible for *Fusarium* toxindegrading activity in a soil Sphingomonas strain. Sci. Rep..

[59] Cobo-díaz JF, Baroncelli R, Floch GL, Picot A (2019). Combined metabarcoding and co-occurrence network analysis to profile the bacterial, fungal and *Fusarium* communities and their interactions in maize stalks. Front. Microbiol..

[60] De Boer W, Wagenaar AM, Klein Gunnewiek PJA, Van Veen JA (2007). In vitro suppression of fungi caused by combinations of apparently non-antagonistic soil bacteria. FEMS Microbiol. Ecol..

[61] Khan MR, Doohan FM (2009). Bacterium-mediated control of Fusarium head blight disease of wheat and barley and associated mycotoxin contamination of grain. Biol. Control.

[62] Król A (2018). Microbiology neutralization of zearalenone using *Lactococcus lactis* and *Bifidobacterium* sp. Anal. Bioanal. Chem..

[63] Jansson JK, Hofmockel KS (2018). The soil microbiome—from metagenomics to metaphenomics. Curr. Opin. Microbiol..

[64] Nasielski J, Earl H, Deen B (2019). Luxury vegetative nitrogen uptake in maize buffers grain yield under post-silking water and nitrogen stress: a mechanistic understanding. Front. Plant Sci..

[65] Chakraborty S, Newton AC (2011). Climate change, plant diseases and food security: an overview. Plant Pathol..

[66] Tola M, Kebede B (2016). Occurrence, importance and control of mycotoxins: a review. Cogent Food Agric..

[67] Chilaka CA, De-Boevre M, Atanda OO, De-Saeger S (2017). The status of fusarium mycotoxins in sub-Saharan Africa: a review of emerging trends and post-harvest mitigation strategies towards food control. Toxins (Basel).

[68] Reid LM, Mather DE, Hamilton RI, Bolton AT (1992). Diallel analysis of resistance in maize to *Fasarium graminearum* infection via the silk. Can. J. Plant Sci..

[69] Saghai-Maroof MA, Soliman KM, Jorgensen RA, Allard RW (1984). Ribosomal DNA spacer-length polymorphisms in barley: mendelian inheritance, chromosomal location, and population dynamics. Proc. Natl. Acad. Sci..

[70] Walters W (2015). Improved bacterial 16S rRNA gene (V4 and V4–V5) and fungal internal transcribed spacer marker gene primers for microbial community surveys. mSystems.

[71] Lundberg DS, Yourstone S, Mieczkowski P, Jones CD, Dangl JL (2013). Practical innovations for high-throughput amplicon sequencing. Nat. Methods.

[72] Bolyen E (2019). Reproducible, interactive, scalable and extensible microbiome data science using QIIME 2. Nat. Biotechnol..

[73] Callahan BJ (2016). DADA2: High resolution sample inference from Illumina amplicon data. Nat. Methods.

[74] Katoh K, Standley DM (2013). MAFFT multiple sequence alignment software version 7: improvements in performance and usability. Mol. Biol. Evol..

[75] Price MN, Dehal PS, Arkin AP (2010). FastTree 2-Approximately maximum-likelihood trees for large alignments. PLoS ONE.

[76] McDonald, D. et al. The Biological Observation Matrix (BIOM) format or: how I learned to stop worrying and love the ome-ome. *Gigascience* 1, 2047-217X-1-7 (2012). 10.1186/2047-217X-1-7 PMC3626512 23587224

[77] McMurdie PJ, Holmes S (2013). Phyloseq: an R package for reproducible interactive analysis and graphics of microbiome census data. PLoS ONE.

[78] Foster ZSL, Sharpton TJ, Grünwald NJ (2017). Metacoder: an R package for visualization and manipulation of community taxonomic diversity data. PLoS Comput. Biol..

[79] Oksanen, A. J. *et al.* vegan: Community Ecology Package. R package version 2.5-7. https://cran.r-project.org/web/packages/vegan/index.html (2020).

[80] Wickham H (2011). Ggplot2. Wiley Interdiscip. Rev. Comput. Stat..

[81] Chen H, Boutros PC (2011). VennDiagram : a package for the generation of highly-customizable Venn and Euler diagrams in R. BMC Bioinform..

[82] Love MI, Anders S, Huber W (2014). Differential analysis of count data - the DESeq2 package. Genome Biol..

[83] Lahti L, Shetty S, Blake T, Salojarvi J (2017). Microbiome R package. Bioconductor.

[84] Schloerke, B. *et al.* GGally: extension to ggplot. R package version 0.5.0. http://CRAN.R-project.org/package=GGally (2014).

[85] Douglas, G. M. *et al.* PICRUSt2: an improved and extensible approach for metagenome inference. bioRxiv (2019). 10.1101/672295.

[86] Kanehisa M, Goto S (2000). KEGG: kyoto encyclopedia of genes and genomes. Nucleic Acids Res..

[87] Kanehisa M (2019). Toward understanding the origin and evolution of cellular organisms. Protein Sci..

[88] Kanehisa M, Furumichi M, Sato Y, Ishiguro-Watanabe M, Tanabe M (2021). KEGG: integrating viruses and cellular organisms. Nucleic Acids Res..

