# Supplementary material for: Transmitting silks of maize have a complex and dynamic microbiome
Source: Sci Rep. 2021 Jun 24;11:13215. doi: 10.1038/s41598-021-92648-4 (PMC8225909; doi:10.1038/s41598-021-92648-4)
Supplement: Supplementary file 1 — Supplementary Information. [file 41598_2021_92648_MOESM1_ESM.pdf]

Transmitting silks of maize have a complex and dynamic microbiome

Eman M. Khalaf[1,2], Anuja Shrestha[1], Jeffrey Rinne [1], D.J. Michael Lynch[3], Charles R. Shearer [1], Victor Limay-Rios[4], Lana M. Reid[5] and Manish N. Raizada[1*]

[1]Department of Plant Agriculture, University of Guelph, 50 Stone Road E, Guelph, Ontario, Canada, N1G 2W1

[2]Department of Microbiology and Immunology, Faculty of Pharmacy, Damanhour University, Damanhour, Egypt, 22511

[3]Metagenom Bio, 550 Parkside Drive, Unit A9, Waterloo, ON, Canada, N2L 5V4

[4]Department of Plant Agriculture, University of Guelph, Ridgetown Campus, 120 Main Street E, Ridgetown, Ontario, Canada, N0P 2C0

[5]Ottawa Research and Development Centre, Agriculture and Agri-Food Canada, 960 Carling Avenue, Central Experimental Farm, Ottawa, Ontario, Canada, K1A 0C6

*Author for correspondence: Manish N. Raizada, Department of Plant Agriculture, University of Guelph, 50 Stone Road E, Guelph, Ontario, Canada, N1G 2W1. Phone: 1-519-824-4120 x53396; Email: raizada@uoguelph.ca

Supplementary Information

Impact of DNA extraction method on DNA quality. For testing the impact of the DNA extraction method on the quality of generated sequences, DNA of 15 samples was purified using both DNA extraction protocols. Samples included healthy and *Fg*-infected silk base tissues. The generated sequences from DNA purified by the kit protocol were significantly higher (593 taxa of 571,371 reads) when compared to those generated from DNA obtained by the CTAB protocol (355 taxa of 286,215 reads) of the same samples (Supplementary Fig. S3a-c). Furthermore, taxa with very low relative abundance were the most dramatically influenced by the DNA purification protocol. Despite base tissue treatment with CTAB negatively impacting the quality of reads, the compositional analyses exhibited higher read counts and diversity compared to the tips which were treated with the kit protocol (Supplementary Fig. S3a-c), though the sequences were filtered at different quality scores (at QS30 for silk tip sequences and at QS25 for silk base sequences).

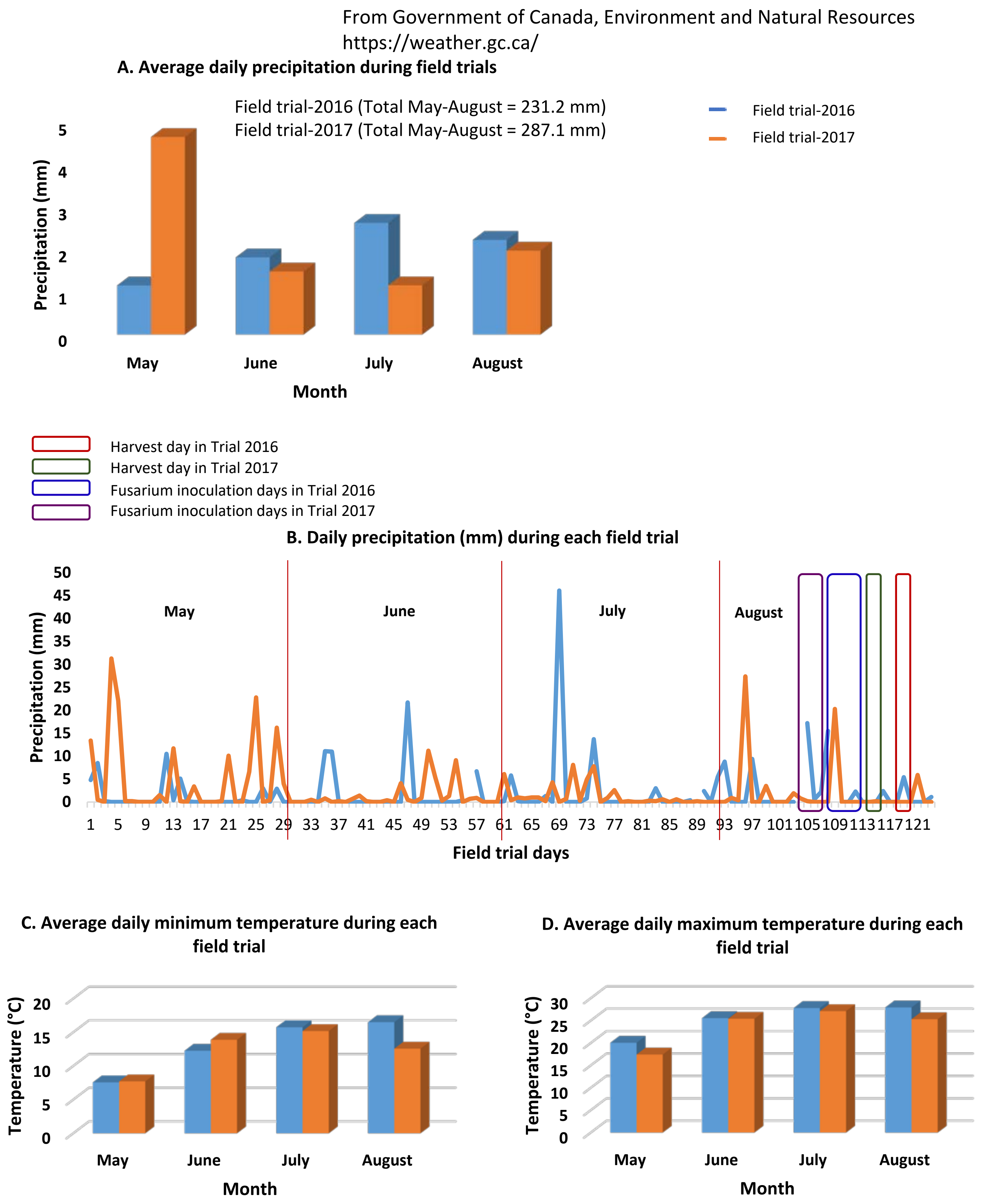

Supplementary Figure S1. Ridgetown field site climate data during experimental field trials showing precipitation and temperature. Graphs (**a-d**) illustrate **(a)** average daily precipitation (mm) by month during the field trials, **(b)** daily precipitation (mm), **(c)** average daily minimum temperature and **(d)** average daily maximum daily temperature. Climate data are from http://climate.weather.gc.ca/

From Government of Canada, Environment and Natural Resources
https://weather.gc.ca/

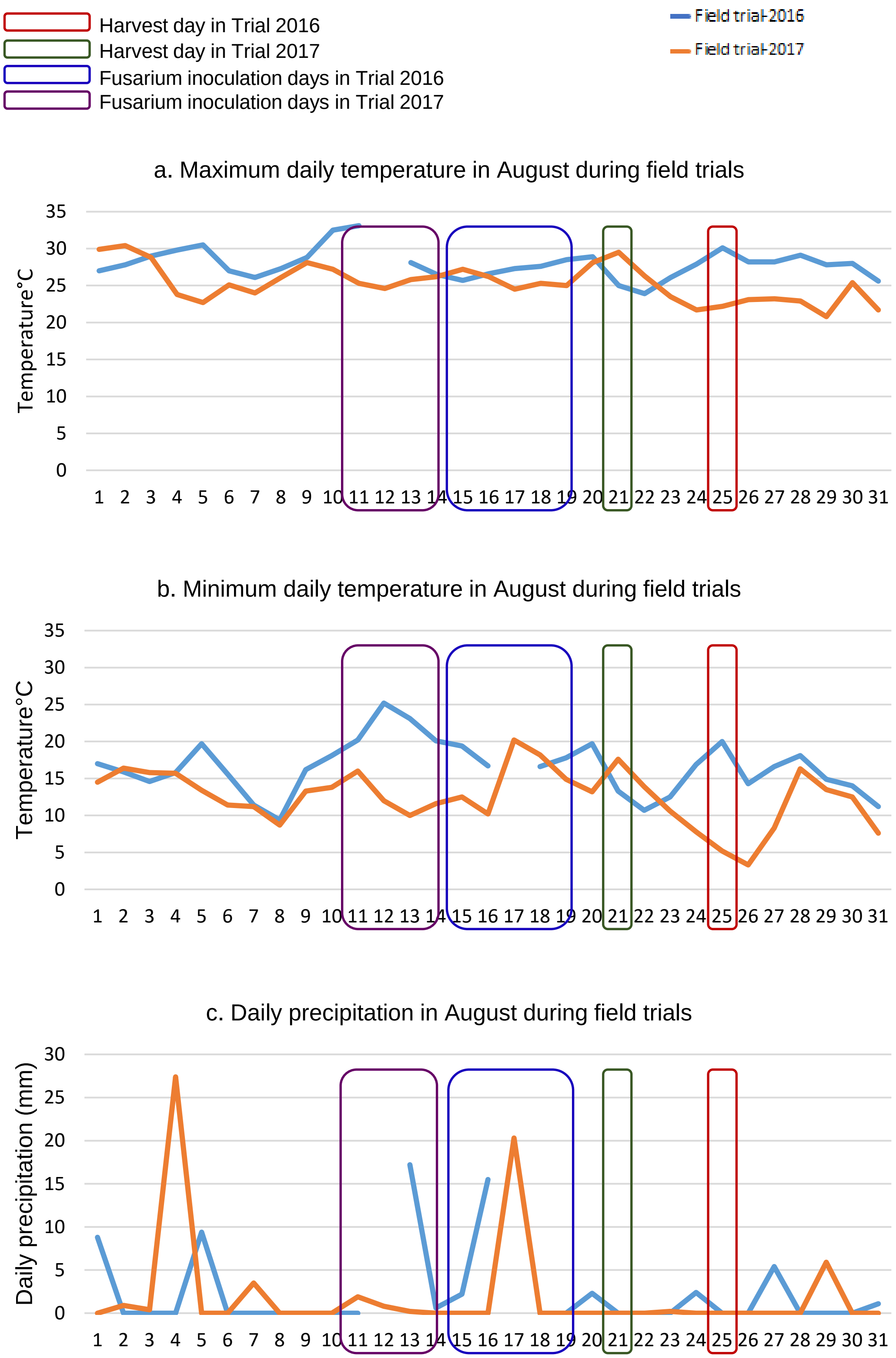

Supplementary Figure S2. Detailed historical climate information at the Ridgetown field site during the critical *Fusarium* inoculation to silk-harvest interval in August of each trial year. Graphs **(a-c)** illustrate **(a)** maximum daily temperature, **(b)** minimum daily temperature, and **(c)** daily precipitation during each field trial. Climate data are from http://climate.weather.gc.ca/

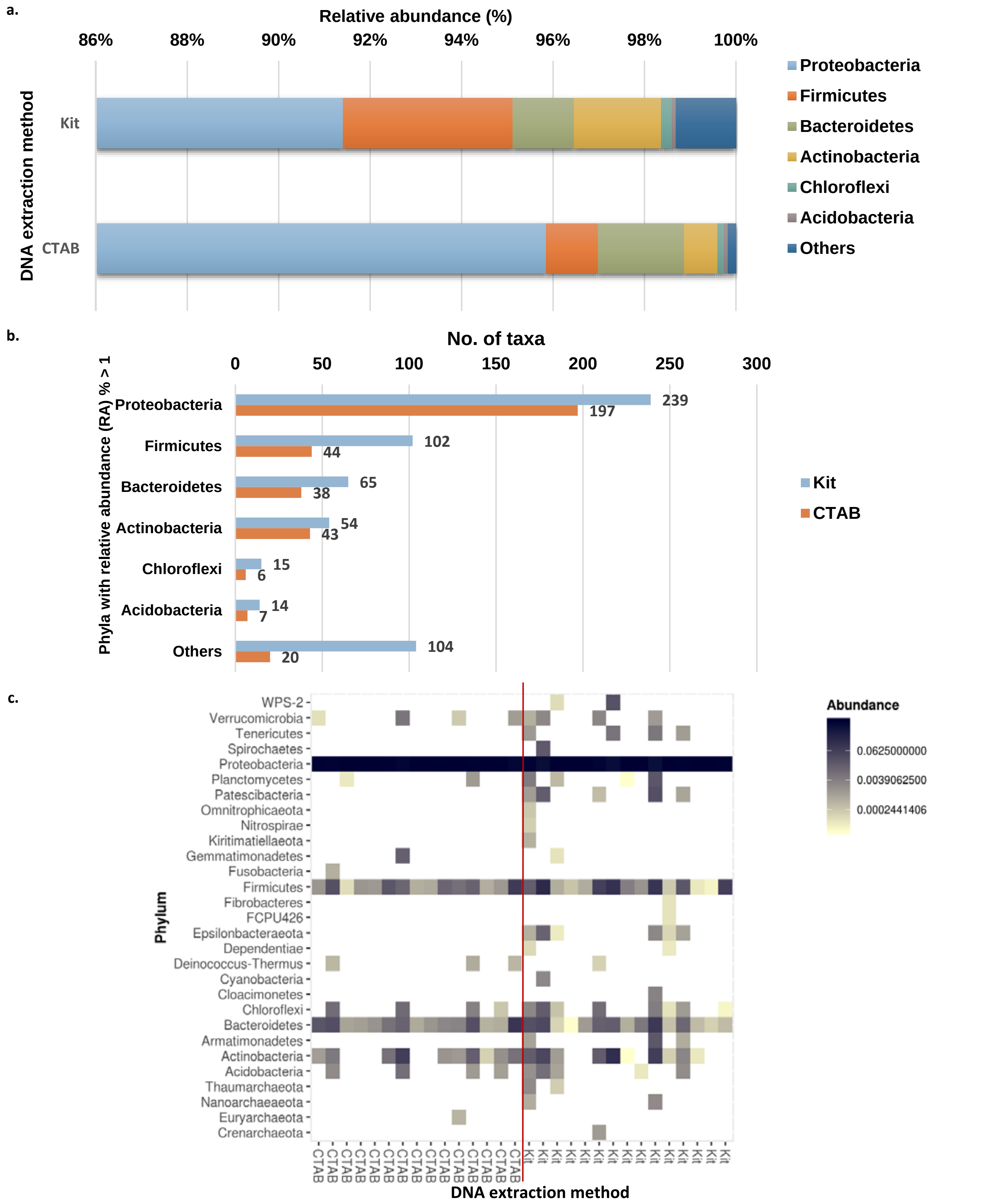

Supplementary Figure S3. The impact of the genomic DNA extraction method on TSM composition. A side experiment (see Appendix S1) was conducted to determine the impact on TSM of extracting genomic DNA using a Qiagen Plant DNeasy Kit (Kit, n=15) compared to CTAB (n=15). **(a)** Bar chart of percent relative abundance (RA) of bacterial phyla within their respective communities (based on 16S read counts) sorted by DNA extraction methods. Phyla with RA < 1% were grouped altogether and labeled as “Others”; (**b)** bar chart displaying the number of taxa in each dominant phylum (with RA>1%) for each DNA extraction method; (**c)** heatmap of TSM at the phylum level, displayed by the DNA extraction method.

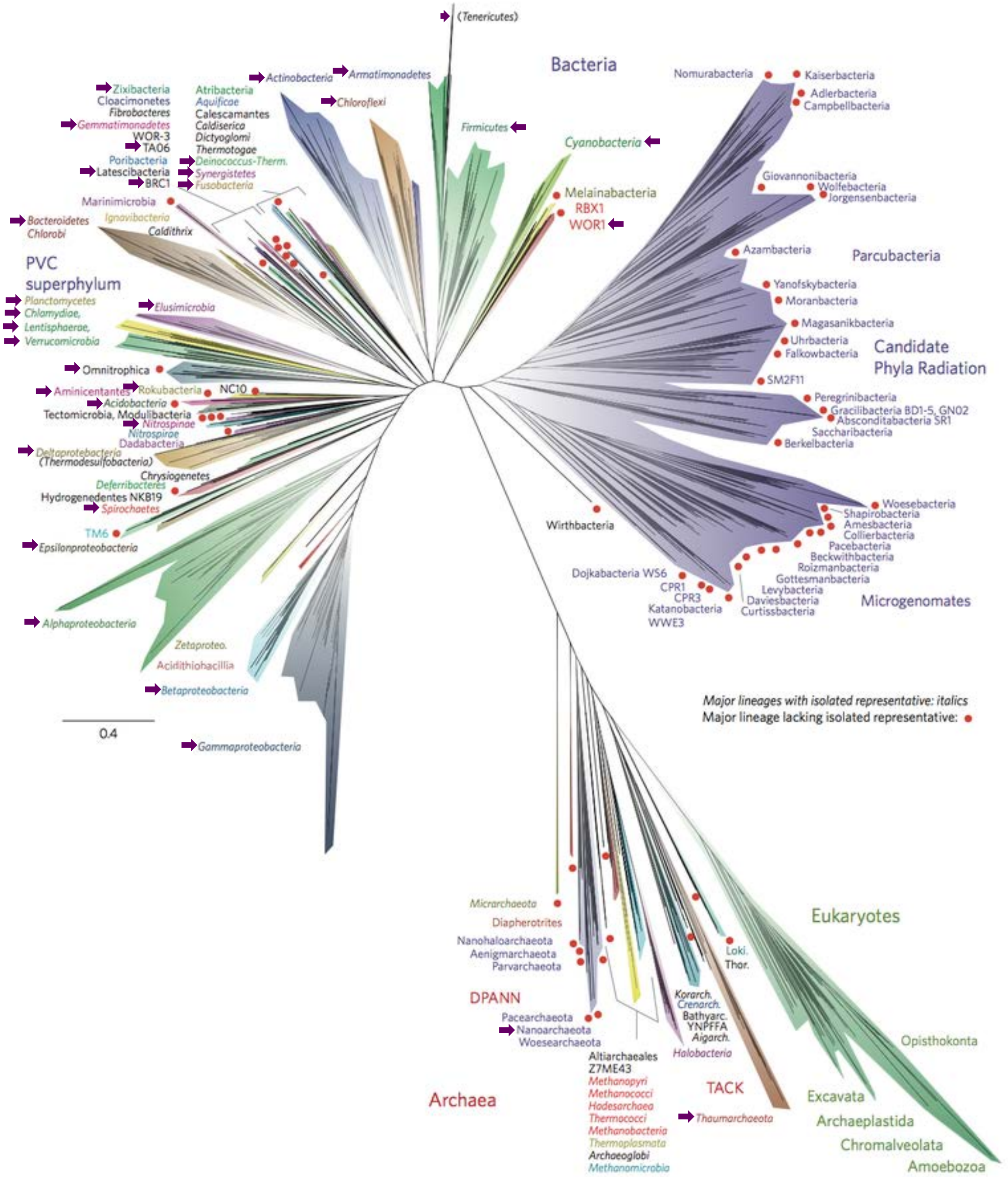

Supplementary Figure S4. Overview of TSM at the highest levels of taxonomic hierarchy. Locations of TSM across the prokaryotic tree of life [23] displayed at domain, phylum and class taxonomic levels.

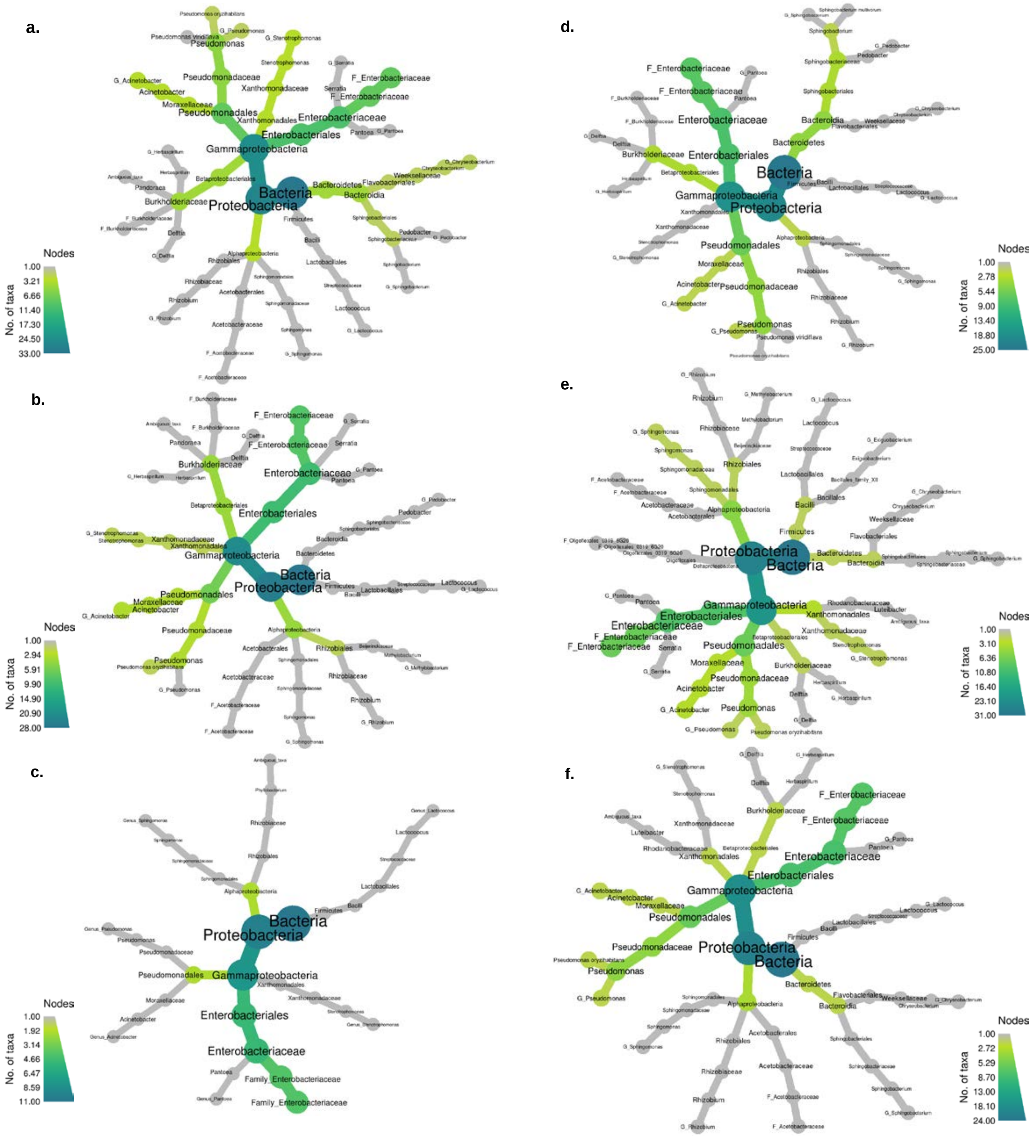

Supplementary Figure S5. Impact of year and *Fg* infection on core TSM of silk tissues. Calculated core taxa from 2016 for **(a)** *Fg*-infected silk tip tissues, and **(b)** *Fg*-infected silk base tissues (data for 2016 healthy tip, and base core TSM are in Fig. 1e,f). Calculated core taxa from 2017 for **(c)** healthy tip tissues. **(d)** *Fg*-infected silk tip tissues, **(e)** healthy base tissues, and **(f)** *Fg*-infected base tissues. Core taxa (prevalent ≥50% of silk samples) are displayed in a hierarchal taxonomic heat tree from kingdom to species. The color depth and node size indicate the number of bacterial taxa within each taxonomic node or branch.

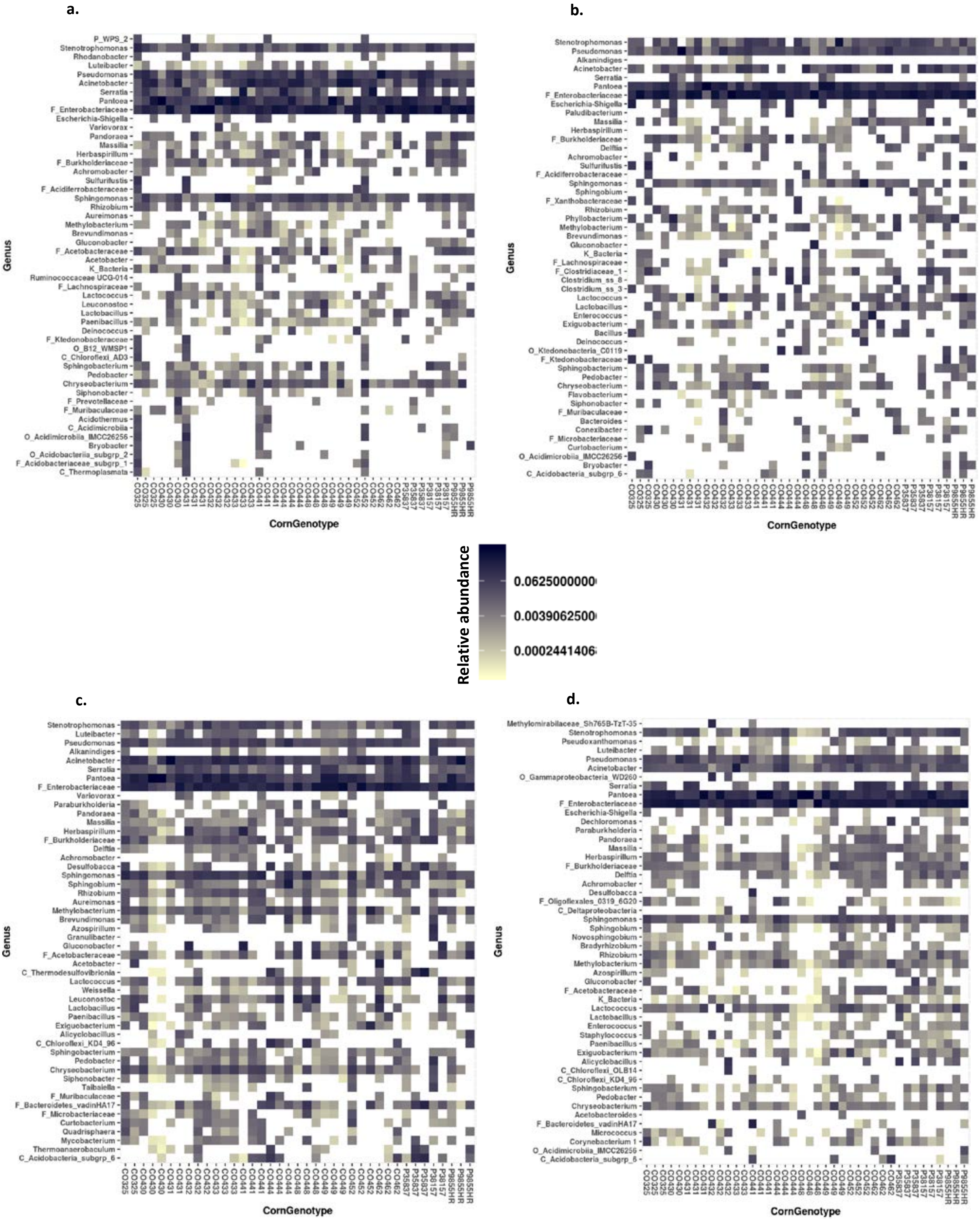

Supplementary Figure S6. Temporal and spatial reproducibility of dominant genera of healthy TSM across all tested maize genotypes. (a, b) Heatmaps display the relative abundance (RA) of the top 50 TSM genera of healthy silk tips in **(a)** 2016, and **(b)** 2017. **(c, d)** Heatmaps display the relative abundance (RA) of the top 50 TSM genera of healthy silk base tissues in **(c)** 2016 and **(d)** 2017. The x-axis represents the different maize host genotype sources.

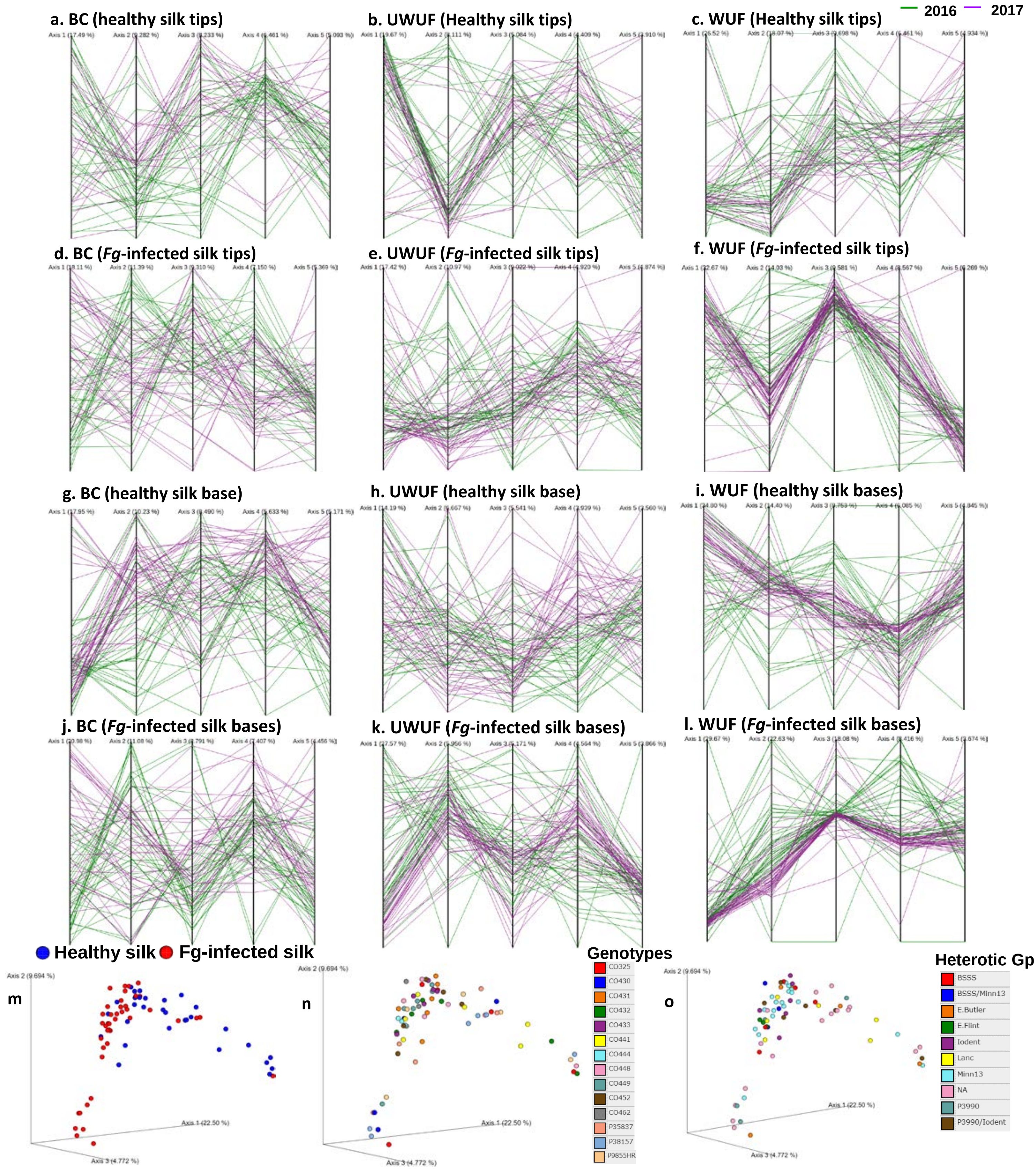

Supplementary Figure S7. Impact of trial year and other factors on TSM composition of healthy and *Fg*-infected tip and base tissues. (a-l) PCoA plots of trial year impact are displayed as 5 principal coordinates (vertical lines) where each horizontal coloured line represents one sample: (**a, d, g, j)** display TSM shifts using the Bray-Curtis (BC) distance matrix calculated on 16S read counts; **(b, e, h, k)** display TSM shifts using the unweighted UniFrac (UWUF) distance matrix that focuses on rare taxa: and **(c, f, i, l)** display TSM shifts using the weighted UniFrac (WUF) distance matrix, that focuses on dominant taxa. **(m-o)** Effect of other factors: PCoA plots showing comparative shifts [unweighted UniFrac (UWUF)] in the TSM of tip 2017 tissues based on **(m)** *Fg* treatment, **(n)** host genotype, and **(o)** host heterotic group.

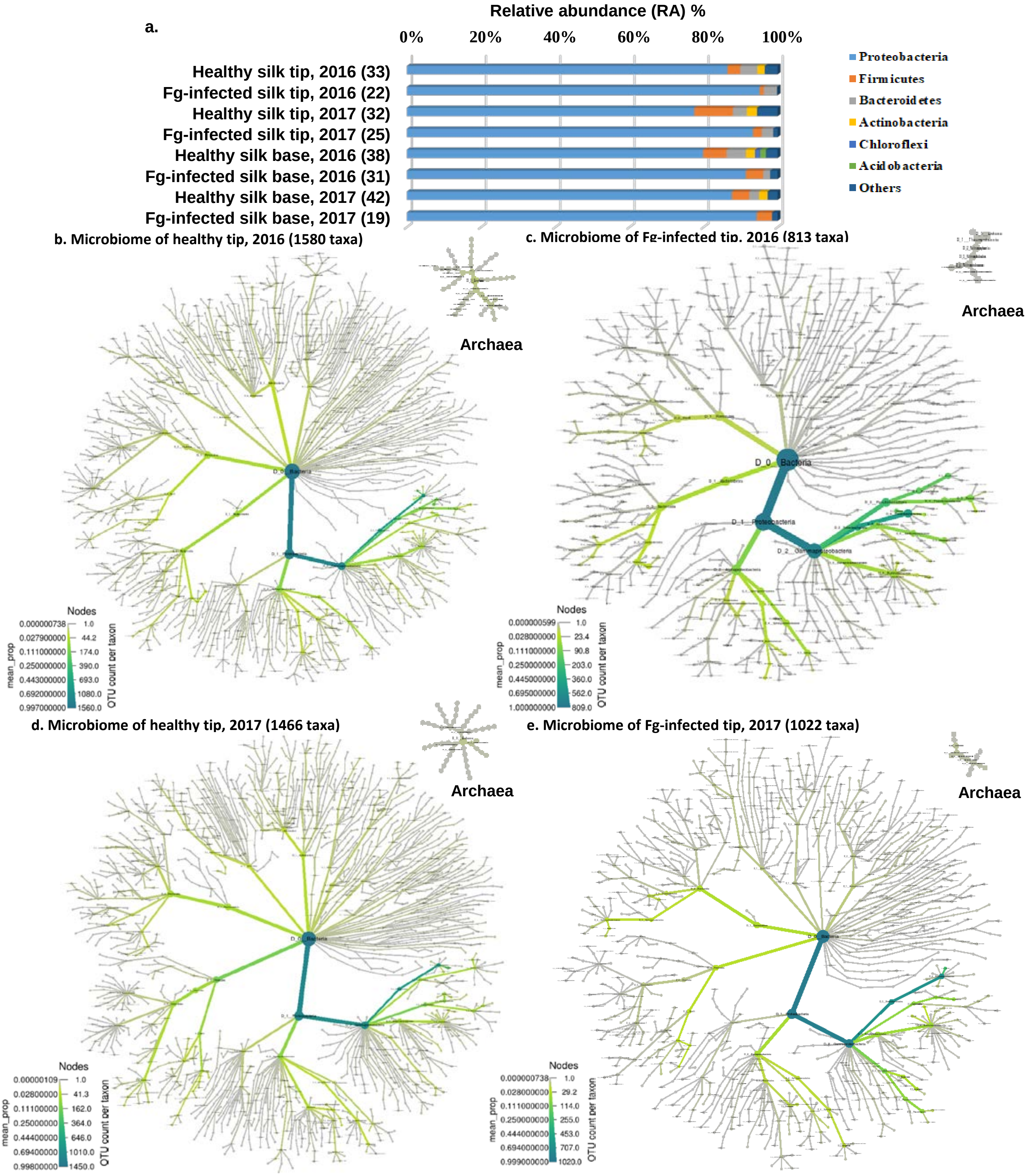

Supplementary Figure S8. Microbiome composition of the TSM of healthy and *Fg*-infected silk tip tissues. **(a)** Bar chart of TSM at the phylum level that demonstrates the contribution of dominant bacterial phyla (relative abundance ≥ 1%) in each treatment group by year. **(b-e)** Hierarchal taxonomic heat trees of total TSM communities from: **(b)** healthy silk tips (2016), **(c)** *Fg*-infected silk tips (2016), **(d)** healthy silk tips (2017), and **(e)** *Fg*-infected silk tips (2017). The data are displayed from kingdom to species. The node sizes indicate the number of bacterial taxa. The node colours indicate the mean proportion of the 16S read counts (calculated by converting the read counts of each taxa into fractions of the total microbial community).

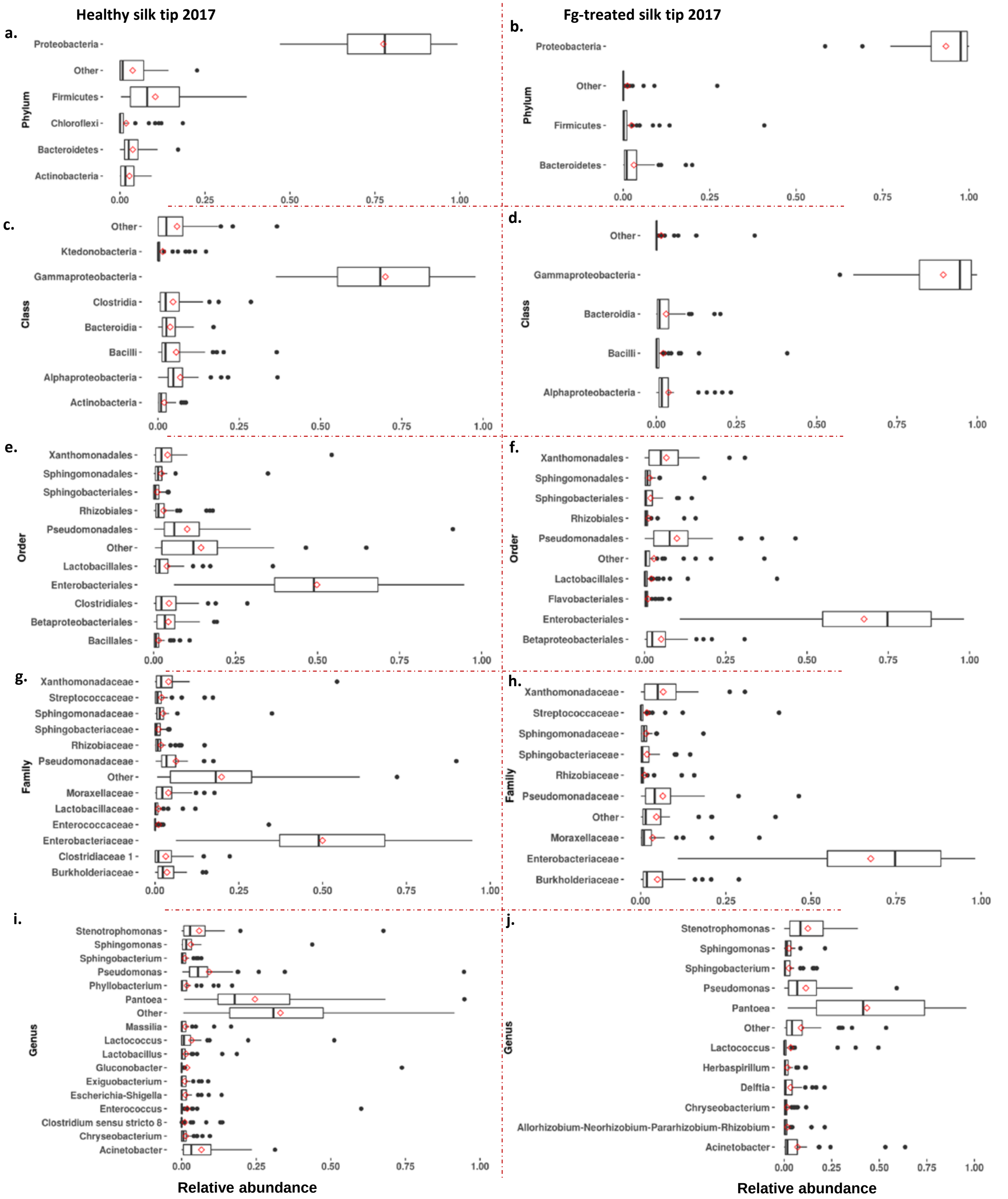

Supplementary Figure S9. Impact of *Fg* infection on mean relative abundance (RA) of TSM dominant taxa of silk tip tissues (2017) calculated at phylum-to-genus taxonomic levels. Boxplots of calculated mean RA at: **(a, b)** phylum level, **(c, d)** class level, **(e, f)** order level, **(g, h)** family level, and **(I, J)** genus level. **(a, c, e, g, i)** are healthy silk tips, and **(b, d, f, h, j)** are *Fg*-infected silk tips.

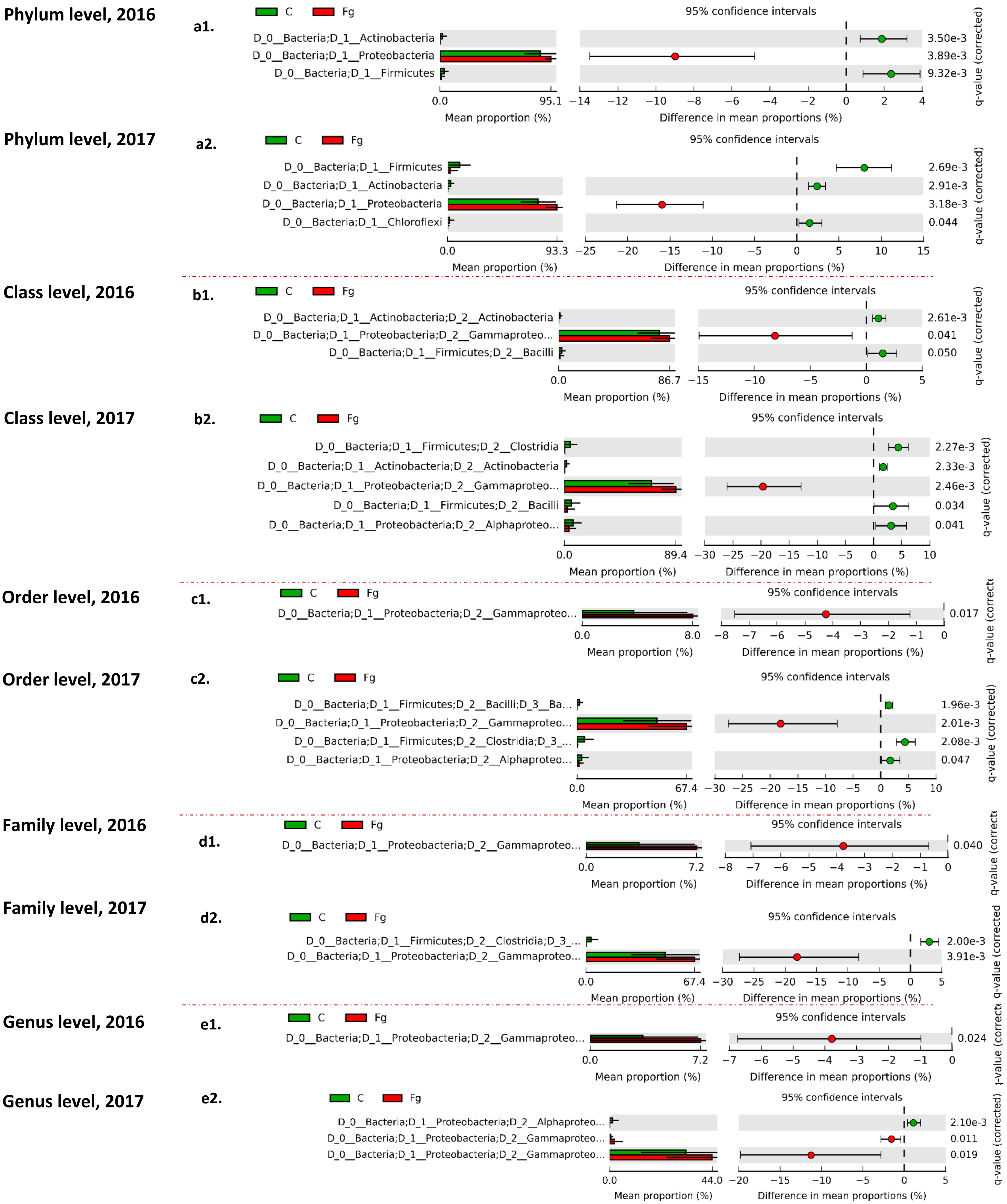

Supplementary Figure S10. Extended error bar (EEB) plots that demonstrate the impact of *Fg* infection and year on TSM taxa of silk tip tissues, at phylum-to-genus taxonomic levels. Statistical calculations and EEB plots were created using STAMP software (see Appendix S1). EEB plots display TSM taxa that significantly changed upon *Fg* infection at: **(a1, a2) the** phylum level, **(b1, b2)** class level**, (c1, c2)** order level, **(d1, d2)** family level, and **(e1, e2)** genus level.

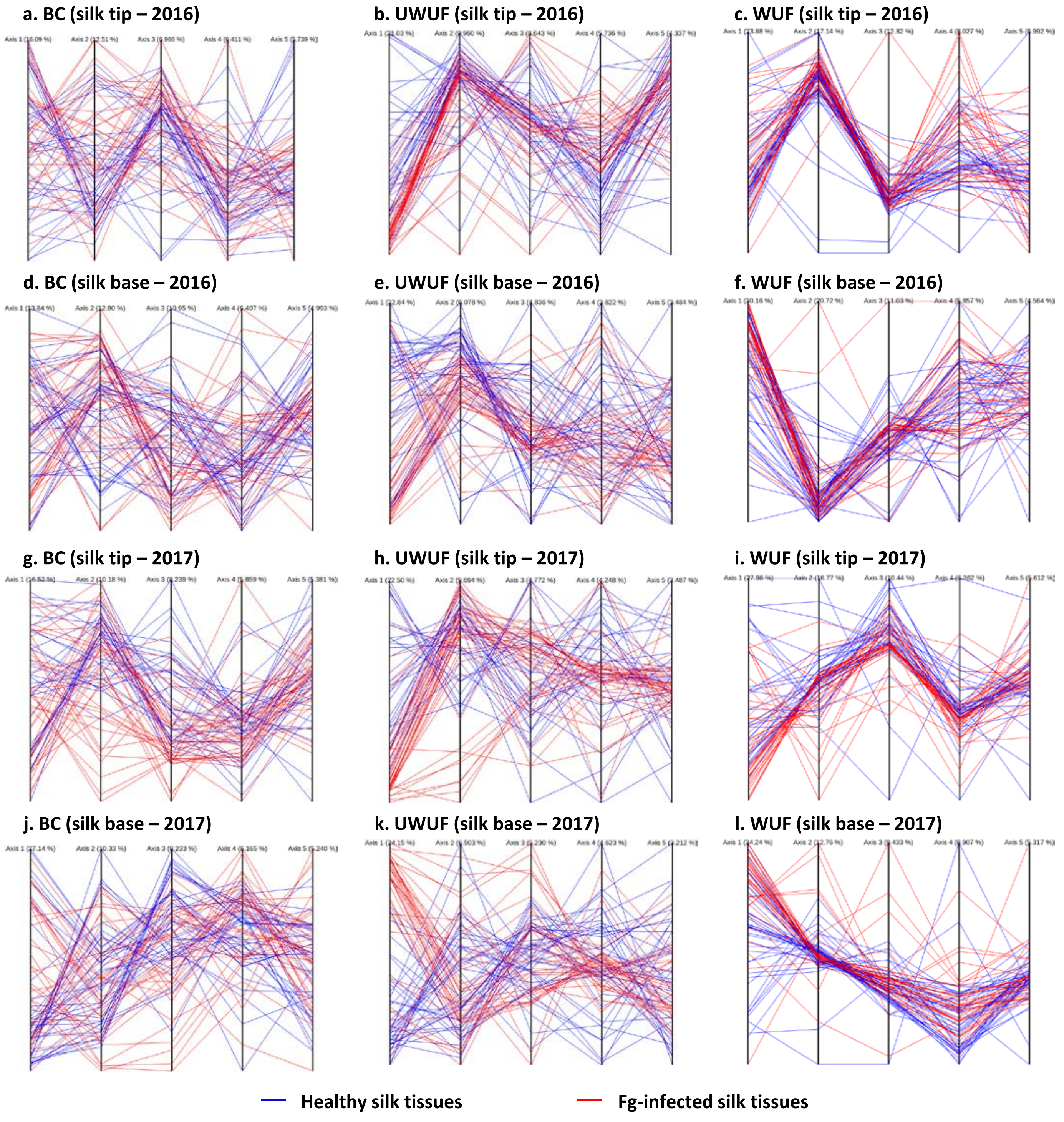

Supplementary Figure S11. Shifts in TSM composition of silk tip and base tissues upon *Fg* infection. PCoA plots are displayed as 5 principal coordinates (vertical lines) where each horizontal coloured line represents one sample: (**a, d, g, j)** display TSM shifts using the Bray-Curtis (BC) distance matrix calculated on 16S read counts; **(b, e, h, k)** display TSM shifts using the unweighted UniFrac (UWUF) distance matrix that focuses on rare taxa: and **(c, f, i, l)** display TSM shifts using the weighted UniFrac (WUF) distance matrix, that focuses on dominant taxa.

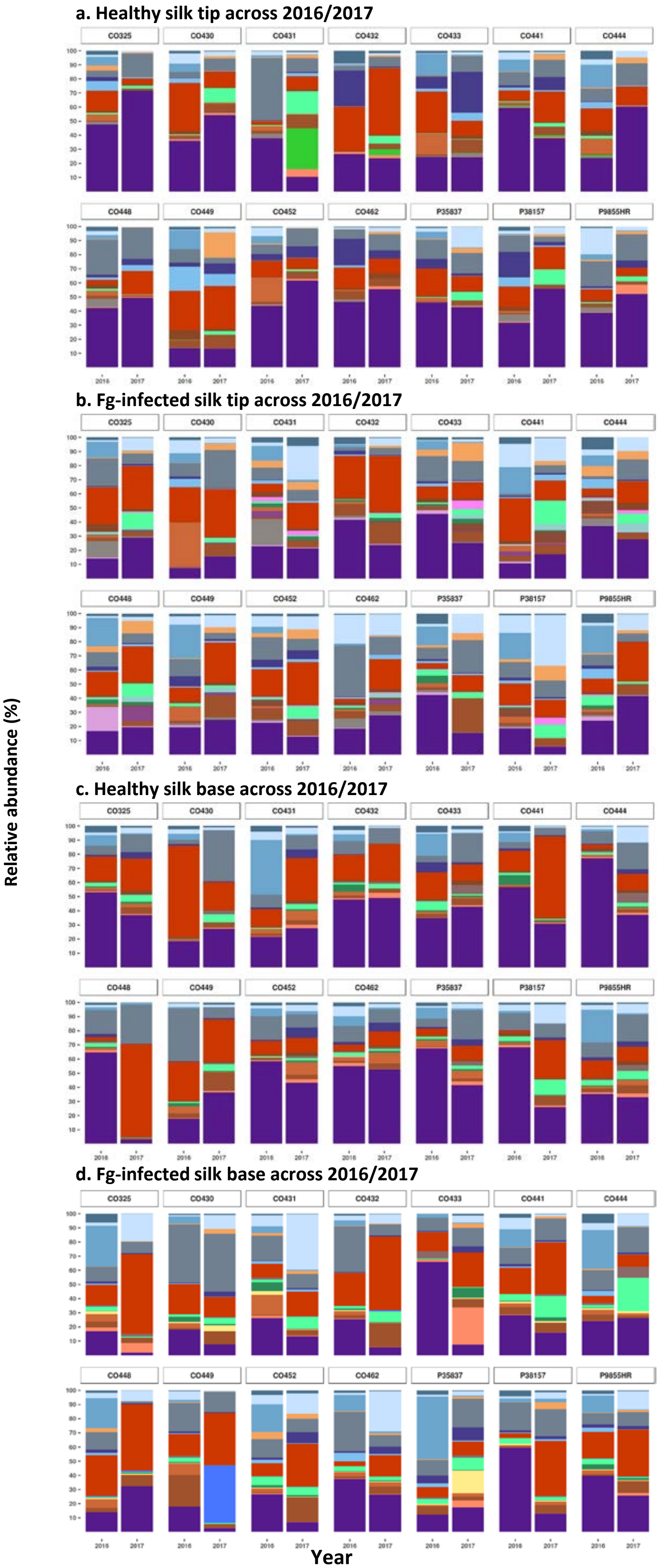

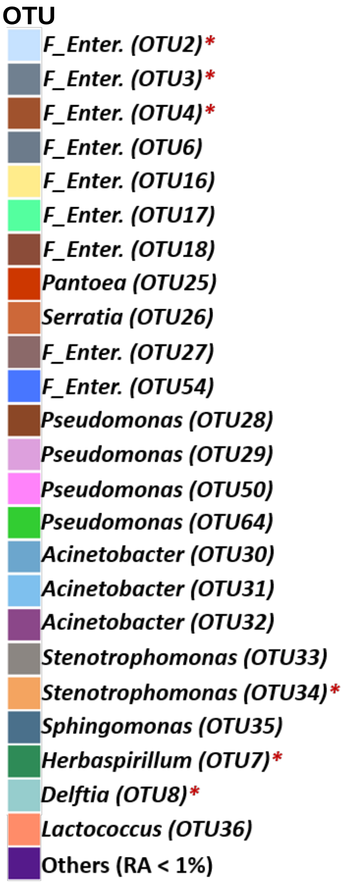

E. List of grown maize genotypes & their susceptibility to Fg via silk

CO325	I
CO430	HR
CO431	R
CO432	HR
CO433	R
CO441	R
CO444	I
CO448	I
CO449	MR-R
CO452	I
CO462	S
P35837	-
P38157	-
P9855HR	-

R: resistant,
I: intermediate,
MR-R: moderately resistant to resistant,
S: sensitive

Supplementary Figure S12. Impact of field year on TSM composition for each maize genotype. (a-d) Bar charts of dominant TSM taxa [relative abundance (RA) ≥1%] of 14 grown maize genotypes in 2016 and 2017 calculated at the OTU level for **(a)** healthy silk tip tissues, **(b)** *Fg*-infected silk tip tissues, **(c)** healthy silk base tissues, **(d)** *Fg*-infected silk tip tissues. OTUs with RA <1% were grouped and labeled as “Others”. **(e)** List of sampled maize genotypes along with their corresponding silk susceptibility to *Fg*.

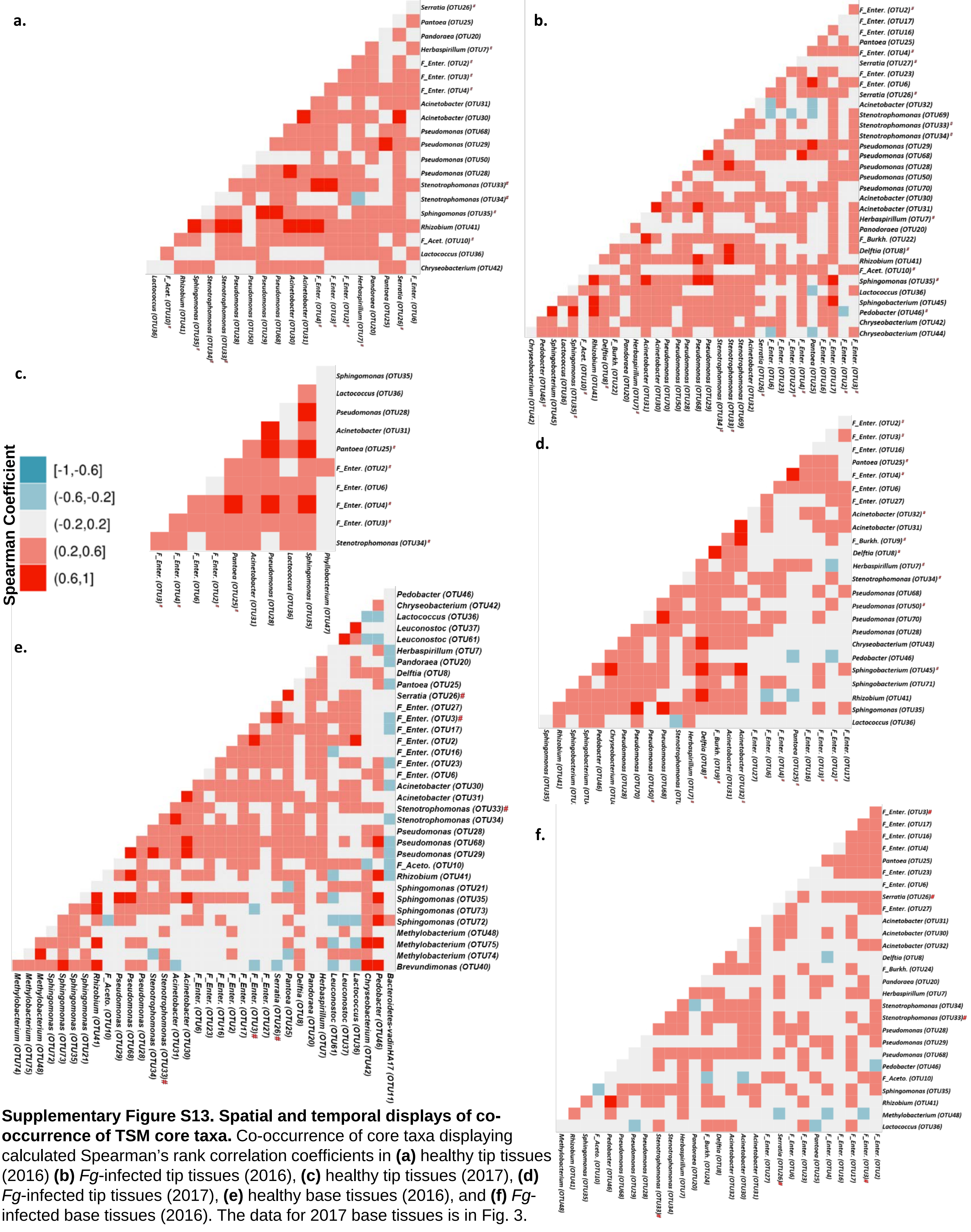

Supplementary Figure S13. Spatial and temporal displays of co-occurrence of TSM core taxa. Co-occurrence of core taxa displaying calculated Spearman's rank correlation coefficients in **(a)** healthy tip tissues (2016) **(b)** *Fg*-infected tip tissues (2016), **(c)** healthy tip tissues (2017), **(d)** *Fg*-infected tip tissues (2017), **(e)** healthy base tissues (2016), and **(f)** *Fg*-infected base tissues (2016). The data for 2017 base tissues is in Fig. 3.

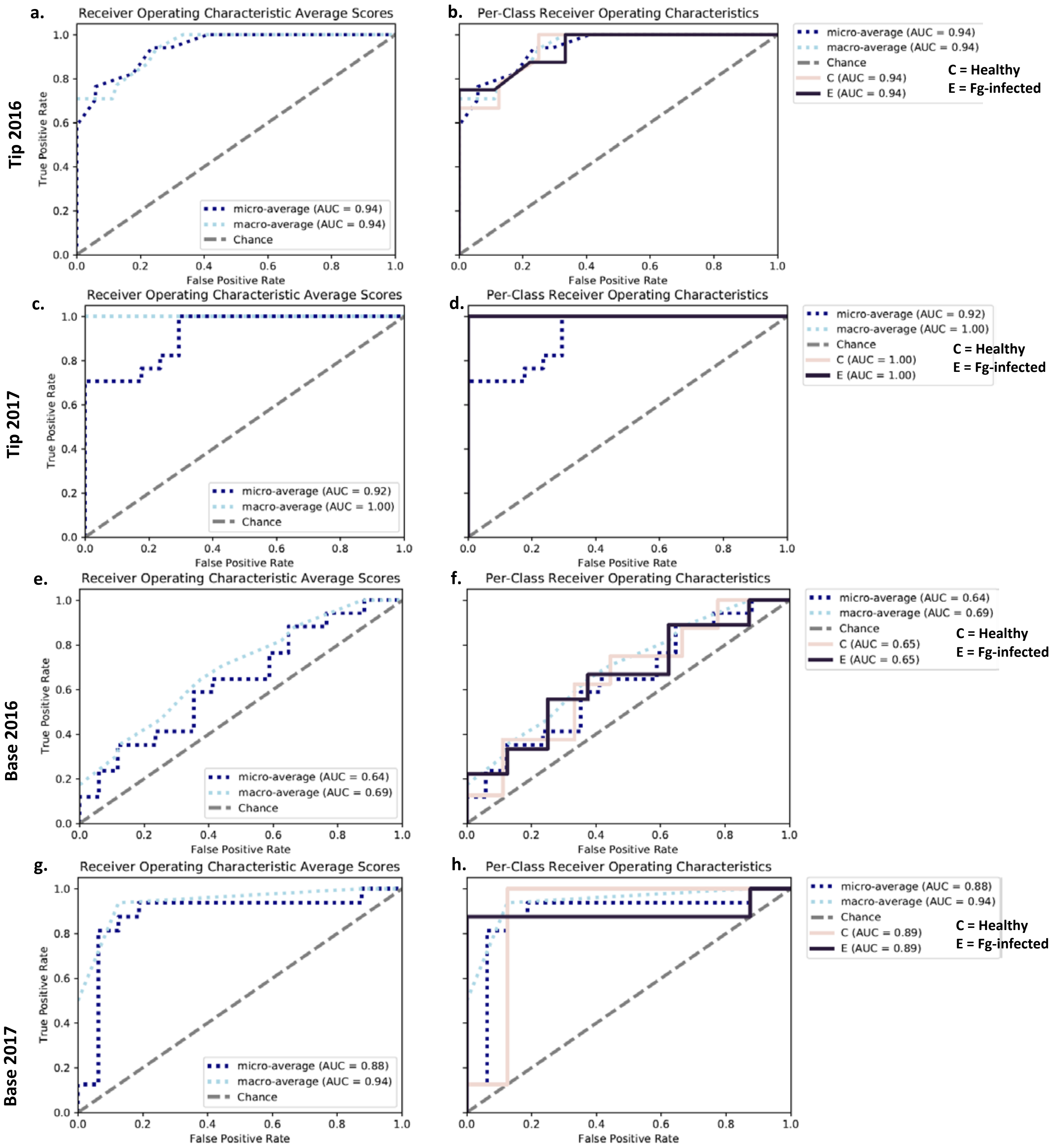

Supplementary Figure S14. Curves of calculated Receiver Operating Characteristic (ROC) average scores and per-class Receiver Operating Characteristics. (a, c, e, g) Curves of calculated ROC for transmitting **(a)** silk tip tissues (2016), **(c)** silk tip tissues (2017), **(e)** silk base tissues (2016), **(g)** silk base tissues (2017). **(b, d, f, h)** Curves of calculated per-class ROC for healthy samples versus *Fg*-infected samples of **(b)** silk tip tissues (2016), **(d)** silk tip tissues (2017), **(f)** silk base tissues (2016), **(h)** silk base tissues (2017). These curves were generated using a supervised machine learning method in Qiime2-2019.10 platform/pipeline. ROC is a probability curve and AUC represents degree or measure of separability. It informs how much a model is capable of distinguishing between treatments. A high AUC means that the model is good. For example, a high AUC means that the model is good at distinguishing between healthy and Fg-infected samples. When the AUC is 0.94, it means there is a 94% chance that the model will be able to distinguish between treatments. When the AUC is approximately 0.5, the model has no discrimination capacity to distinguish between treatments.

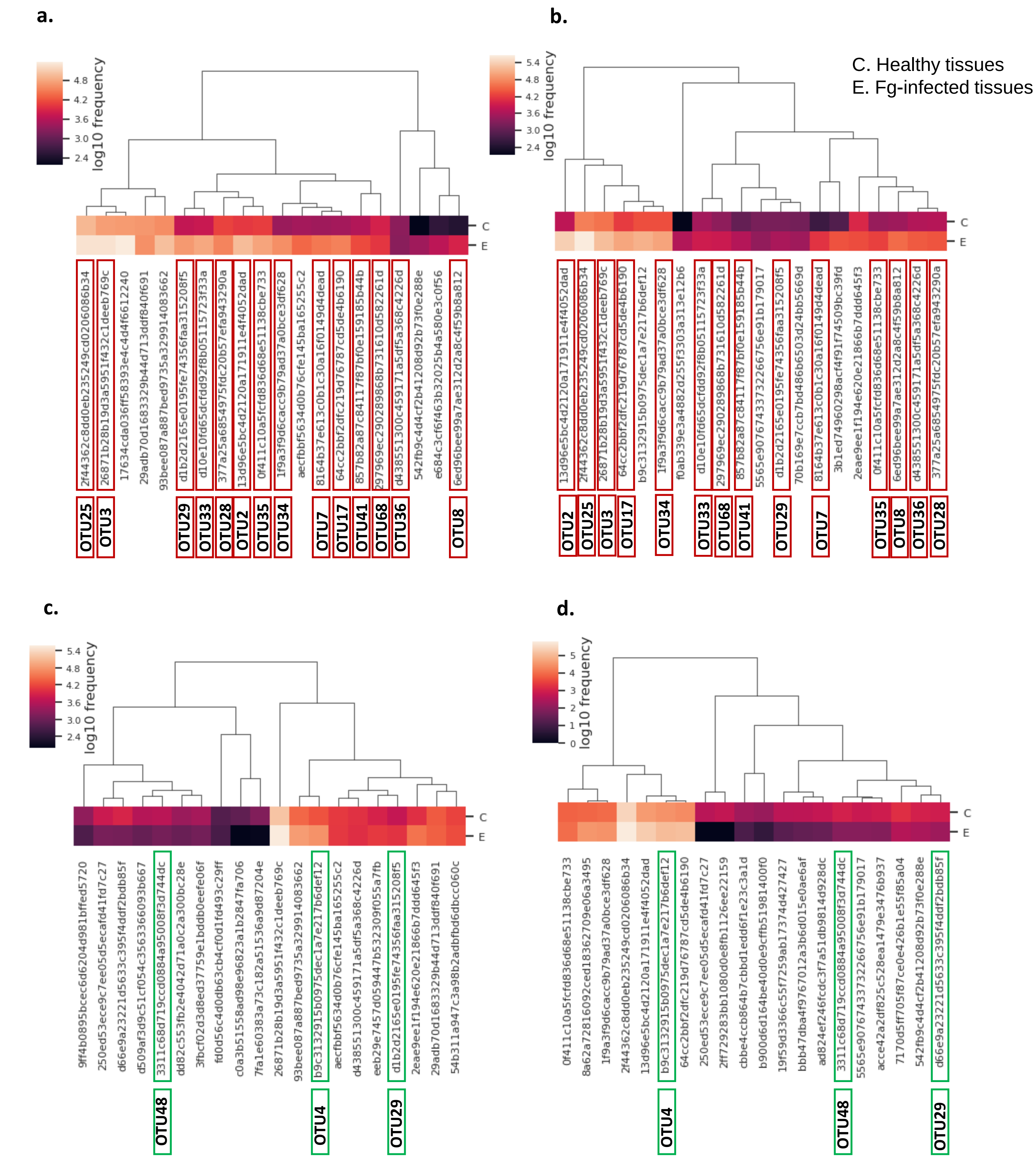

Supplementary Figure S15. Heatmap dendograms of the most important taxa that distinguish between healthy and *Fg*-infected transmitting silk tissues and calculated using the machine-learning prediction of sample data in the Qiime2 platform. (a, b) Heatmap dendograms that display the log10 change in abundance of the top 20 taxa between healthy silk tip tissues and *Fg*-infected silk tip tissues in **(a)** 2016 and **(b)** 2017. **(c, d)** Heatmap dendograms that display the log10 change in abundance of the top 20 taxa between healthy silk base tissues and *Fg*-infected silk base tissues in **(c)** 2016 and **(d)** 2017.

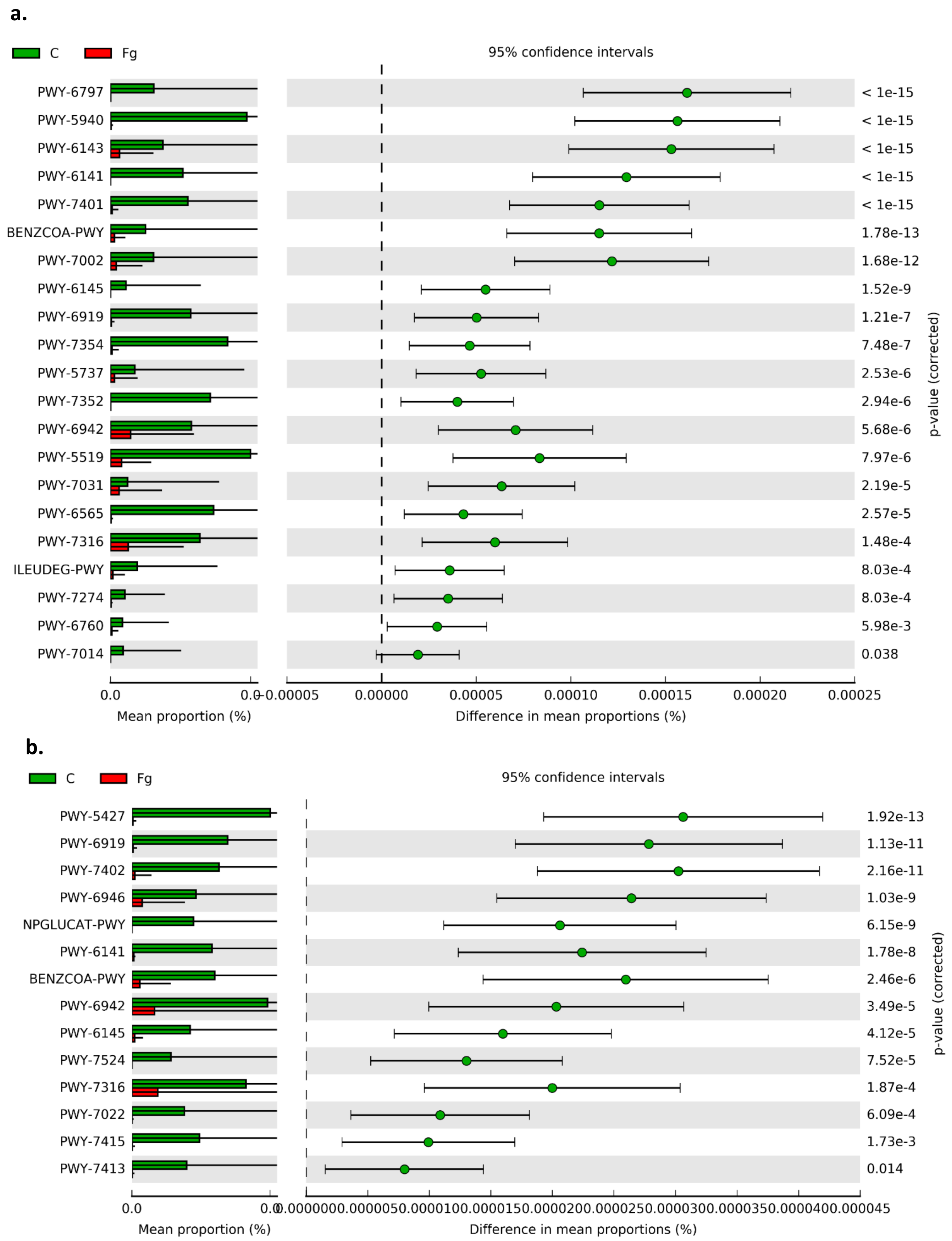

Supplementary Figure S16. Impact of *Fg* infection on predicted TSM metabolic pathways (2017). Statistical calculations and EEB plots were created using STAMP software. Extended error bar (EEB) plots demonstrate predicted TSM metabolic pathways that are significantly impacted upon *Fg* infection in **(a)** silk tip tissues, and **(b)** silk base tissues.

Supplementary Table S1. Summary of the pedigrees of the host maize genotypes tested in this study from the Agriculture and Agri-Food Canada breeding program (Ottawa Research and Development Centre, Canada).

Inbred	Year released	Derivation	Heterotic group	Days to silking	No. of leaves	Ear length (cm)	Ear diameter (cm)	Number of kernel rows	*Gibberella ear rot/ (silk)	*Gibberella ear rot/ (kernel)
**CO462**	2016	CO388 x W153R	BSSS/ Minnesota 13	75	16-18	14-15	4	14-16	S	S
**CO452**	2014	(CO388xCO328)xCO388(4)	BSSS	80	14	18-19	4.5	14	I	I
**CO444**	2007	S1381xCO382	E.Flint	79	17-18	13	4	16-18	I	I
**CO448**	2012	CO273xCO431	P3990/Iodent	70	11-12	12	4	16	I	I
**CO325**	1991	(CO256 X CO264) CO264 (2)	E.Butler	76	17-18	12	3	12	I	I
**CO449**	2012	CO432xCO433	Minnesota 13	75	11-12	14	4.5	16	MR-R	MR-R
**CO441**	2002	Jacques 7700 x CO298	Lancaster	72	13-14	11-13	3	14	R	R
**CO431**	1999	Fusarium Resistant Synthetic	Iodent	71	16-17	14	4	16-18	R	I
**CO433**	2000	Pride K127	Minnesota13	77	15-16	13-14	3	12-13	R	R
**CO430**	1999	Fusarium Resistant Synthetic	P3990	69	14-16	13	4	16	HR	HR
**CO432**	2000	Fusarium Resistant Synthetic C1	Minnesota13	74	16-18	12-14	3	14	HR	I
**P35837**	NA	NA	NA	NA	NA	NA	NA	NA	NA	NA
**P38157**	NA	NA	NA	NA	NA	NA	NA	NA	NA	NA
**P9855HR**	NA	NA	NA	NA	NA	NA	NA	NA	NA	NA

*Abbreviations: S = sensitive; I=intermediate; MR=moderately resistant; R=resistant; HR = highly resistant. NA = Not Available

Supplementary Table S2. Summary of number of samples, number of taxa and number of 16S read counts and associated calculations in each treatment group.

Group	No. of samples	No. of taxa	Total read count	Min read count/sample	Max read count/sample	Median read count/sample	Mean read count/sample
**A**	42	1580	514,304	417	64,546	6,002	12,245.33
**B**	41	813	1,726,101	50	103,390	39,418	42,100.02
**C**	42	1466	318,717	388	43,775	2,492.20	7,588.50
**D**	42	1022	1,734,839	2,127	251,727	40,101.50	41,305.69
**E**	39	1677	1,198,548	3,248	89,755	26,567	30,732
**F**	42	1339	1,684,800	9,762	75,010	41,663.50	40,114.29
**G**	40	1547	837,935	2,881	61,841	16,328.50	20,948.38
**H**	40	629	1,830,315	7,286	83,023	49,410.50	45,757.88

***Sample groupings:**
A. Healthy tip 2016
B. Fusarium-infected tip 2016
C. Healthy tip 2017
D. Fusarium -infected tip 2017
E. Healthy base 2016
F. Fusarium-infected base 2016
G. Healthy base 2017
H. Fusarium-infected base 2017

Sequence length statistics for the silk tip tissues

Sequence Count	Min Length	Max Length	Mean Length	Range	Standard Deviation
4149	228	359	253.79	131	4.54

Seven-Number summary of sequence length of the silk tip tissues

**Percentile:**	2%	9%	25%	50%	75%	91%	98%
**Length* (nts):**	246	253	254	254	254	254	255

Sequence length statistics for the silk base tissues

Sequence Count	Min Length	Max Length	Mean Length	Range	Standard Deviation
**4214**	228	338	253.8	110	4.68

Seven-Number summary of sequence length of the silk base tissues

**Percentile:**	2%	9%	25%	50%	75%	91%	98%
**Length* (nts):**	249	254	254	254	254	254	255

Supplementary Table S3. Summary of mean relative abundance of dominant (>1%) TSM taxa in silk tip (Sheet S3a), and base tissues (Sheet S3b).

Supplementary Table S3a: Summary of mean relative abundance of dominant (>1%) TSM taxa in silk tip tissues.

Taxonomic level	Taxon	Healthy tip (2016)	Fg-infected tip (2016)	Healthy tip (2017)	Fg-infected tip (2017)
**Class**		N= 73	N= 44	N= 81	N= 49
	Gammaproteobacteria	78.76	86.65	69.94	89.42
	Alphaproteobacteria	7.23	8.46	6.86	3.8
	Bacteroidia	4.49	3.55	3.75	3.07
	Bacilli	2.56	1.07	5.6	2.16
	Actinobacteria	1.1	<1.0	1.93	<1.0
	Clostridia	<1.0	<1.0	4.61	<1.0
	Ktedonobacteria	<1.0	<1.0	1.42	<1.0
**Family**		N= 271	N= 129	N= 307	N= 178
	Enterobacteriaceae	51.64	49.48	49.95	67.57
	Moraxellaceae	10.49	15.54	3.97	3.48
	Pseudomonadaceae	7.32	9.05	6.26	6.46
	Burkholderiaceae	4.06	4.52	3.56	4.9
	Sphingomonadaceae	3.61	4.21	2.37	1.57
	Xanthomonadaceae	3.51	7.22	4.02	6.59
	Weeksellaceae	2.25	2.14	<1.0	<1.0
	Acetobacteraceae	1.76	2.98	<1.0	<1.0
	Sphingobacteriaceae	<1.0	1.36	1.04	1.84
	Clostridiaceae 1	<1.0	<1.0	3.18	<1.0
	Enterococcaceae	<1.0	<1.0	1.04	<1.0
	Lactobacillaceae	<1.0	<1.0	1	<1.0
	Rhizobiaceae	<1.0	<1.0	1.79	1.17
	Streptococcaceae	<1.0	<1.0	1.93	1.79
**Genus**		N= 455	N= 214	N= 531	N= 311
	Pantoea	24.47	21.63	24.58	43.49
	Acinetobacter	14.58	20.71	6.63	7.06
	Pseudomonas	10.81	13.18	9.21	11.38
	Serratia	6.09	6.73	<1.0	<1.0
	Stenotrophomonas	5.81	10.77	5.88	12.47
	Sphingomonas	4.82	5.98	3.09	2.55
	Chryseobacterium	3.06	3.34	1.39	1.51
	Pandoraea	2.05	2	<1.0	<1.0
	Ambiguous_taxa	1.95	2.48	<1.0	<1.0
	Herbaspirillum	1.6	2.08	<1.0	1.74
	Lactococcus	1.26	<1.0	3.36	3.42
	Sphingobacterium	1.03	1.25	1.09	2.52
	Leuconostoc	1.01	<1.0	<1.0	<1.0
	Acetobacter	<1.0	1.87	<1.0	<1.0
	Rhizobium*	<1.0	1.29	<1.0	1.63
	Clostridium ss 8**	<1.0	<1.0	1.04	<1.0
	Enterococcus	<1.0	<1.0	1.89	<1.0
	Escherichia-Shigella	<1.0	<1.0	1.29	<1.0
	Exiguobacterium	<1.0	<1.0	1.05	<1.0
	Gluconobacter	<1.0	<1.0	1.81	<1.0
	Lactobacillus	<1.0	<1.0	1.49	<1.0
	Massilia	<1.0	<1.0	1.27	<1.0
	Phyllobacterium	<1.0	<1.0	1.75	<1.0
	Delftia	<1.0	<1.0	<1.0	3.31
**Taxa**		N= 1580	N= 813	N= 1466	N= 1022

***Allorhizobium-Neorhizobium-Pararhizobium-Rhizobium** ****Clostridium sensu stricto 8**

Supplementary Table S3b: Summary of mean relative abundance of dominant (>1%) TSM taxa in silk base tissues.

Taxonomic level	Taxon	Healthy base (2016)	Fg-infected base (2016)	Healthy base (2017)	Fg-infected base (2017)
**Class**		N= 91	N= 76	N= 90	N= 36
	Gammaproteobacteria	62.24	81.3	79.69	89.72
	Alphaproteobacteria	15.16	9.44	6.68	4.59
	Bacteroidia	5.13	1.85	2.6	<1.0
	Bacilli	5.68	4.53	4.03	3.92
	Actinobacteria	1.97	<1.0	1.87	<1.0
	Deltaproteobacteria	2.48	<1.0	1.28	<1.0
**Family**		N= 291	N= 271	N= 341	N= 134
	Enterobacteriaceae	40.18	52.46	63.87	81.98
	Moraxellaceae	10.73	16.95	3.63	1.29
	Pseudomonadaceae	3.27	3.26	4.01	2.18
	Burkholderiaceae	5.3	4.57	4.01	1.8
	Sphingomonadaceae	6.34	3.48	2.79	<1.0
	Xanthomonadaceae	2.05	2.92	2.96	2.06
	Weeksellaceae	1.6	<1.0	<1.0	<1.0
	Acetobacteraceae	4.32	3.92	<1.0	3.29
	Bacteroidetes vadinHA17	1.16	<1.0	<1.0	<1.0
	Beijerinckiaceae	1.99	<1.0	<1.0	<1.0
	Leuconostocaceae	2.77	2.33	<1.0	<1.0
	Syntrophaceae	1.21	<1.0	<1.0	<1.0
	Rhizobiaceae	1.37	<1.0	<1.0	<1.0
	Streptococcaceae	1.04	<1.0	1.75	2.61
	Lactobacillaceae	<1.0	1.12	<1.0	<1.0
**Genus**		N= 505	N= 426	N= 547	N= 223
	Pantoea	21.6	22.91	37.62	61.27
	Acinetobacter	14.08	25.26	6.64	2.99
	Pseudomonas	4.41	6.3	7.2	5.65
	Serratia	3.2	8.69	5.13	1.76
	Stenotrophomonas	2.58	4.53	4.8	5.27
	Sphingomonas	6.04	4.89	4.24	2.05
	Chryseobacterium	1.94	1.19	1.18	<1.0
	Pandoraea	1.05	1.26	<1.0	<1.0
	Ambiguous_taxa	1.8	<1.0	<1.0	<1.0
	Herbaspirillum	1.93	2.09	1.43	2.28
	Lactococcus	1.6	1.14	2.69	3.83
	Methylobacterium	2.48	<1.0	1.29	<1.0
	Leuconostoc	3.95	2.49	<1.0	<1.0
	Acetobacter	1.05	1.85	<1.0	<1.0
	Rhizobium*	1.19	1.07	1.24	<1.0
	Sphingobium	1.66	<1.0	<1.0	<1.0
	Desulfobacca	1.16	<1.0	<1.0	<1.0
	Gluconobacter	2.26	1.51	<1.0	4.21
	Delfitia	<1.0	<1.0	1.22	<1.0
	Ambiguous_taxa	<1.0	1.7	<1.0	<1.0
	Lactobacillus	<1.0	1.2	<1.0	<1.0
	Luteibacter	<1.0	1.43	<1.0	<1.0
	Alicyclobacillus	<1.0	<1.0	1.23	<1.0
	Exiguobacterium	<1.0	<1.0	1.04	<1.0
	Escherichia-Shigella	<1.0	<1.0	<1.0	1.48
**Taxa**		N= 1677	N= 1339	N= 1547	N= 629

Supplementary Table S4. Estimates of alpha diversity indices [richness (Observed OTUs], diversity (Shannon), evenness (Pielou index), and Faith's phylogenetic diversity (FPD)] along with their degree of significance for (S4A) silk tip tissues, and (S4B) silk base tissues

Comparison groups	Observed OTUs	Shannon index	Pielou index	Faith's phylogenetic diversity (FPD)
**Supplementary Table S4a**. Silk tip estimates of alpha diversity indices.				
**2016 Trial**				
**Tip tissues**				
a. Healthy vs Fg-infected	0.94 (ns)	0.04 (*)	0.04 (*)	0.002 (**)
b. Healthy vs healthy (replicates)	0.89 (ns)	0.24 (ns)	0.22 (ns)	0.53 (ns)
a. Fg-infected vs Fg-infected (replicates)	0.065 (ns)	0.14 (ns)	0.28 (ns)	0.54 (ns)
**2017 Trial**				
**Tip tissues**				
a. Healthy vs Fg-infected	0.36 (ns)	0.0001 (****)	0.000004 (****)	0.04 *)
b. Healthy vs healthy (replicates)	0.17 (ns)	0.38 (ns)	0.95 (ns)	0.14 (ns)
c. Fg-infected vs Fg-infected (replicates)	0.14 (ns)	0.072 (ns)	0.13 (ns)	0.005 (**)
**Healthy silk tissues (year-to-year)**				
a. Healthy tip 2016 vs healthy tip 2017	0.22 (ns)	0.46 (ns)	0.11 (ns)	0.92 (ns)
**Fg-infected tissues (year-to-year)**				
a. Fg-infected tip 2016 vs Fg-infected tip 2017	0.76 (ns)	0.10 (ns)	0.036 (*)	0.92 (ns)

Supplementary Table S4b. Corresponding silk base estimates of alpha diversity indices.

Comparison groups	Observed OTUs	Shannon index	Pielou index	Faith's phylogenetic diversity (FPD)
**2016 Trial**				
**Base tissues**				
a. Healthy vs Fg-infected	0.0003 (***)	0.0006 (***)	0.004 (**)	0.00007 (****)
b. Healthy vs healthy (replicates)	0.88 (ns)	0.21 (ns)	0.069 (ns)	0.78 (ns)
c. Fg-infected vs Fg-infected (replicates)	0.90 (ns)	0.51 (ns)	0.62 (ns)	0.70 (ns)
**2017 Trial**				
**Base tissues**				
a. Healthy vs Fg-infected	9.37e-7 (****)	0.00019 (***)	0.0014 (**)	4.36e-9 (****)
b. Healthy vs healthy (replicates)	0.002 (**)	0.98 (ns)	0.36 (ns)	0.044 (*)
c. Fg-infected vs Fg-infected (replicates)	0.90 (ns)	0.95 (ns)	0.96 (ns)	0.94 (ns)
**Healthy silk tissues (year-to-year)**				
b. Healthy base 2016 vs healthy base 2017	0.033 (*)	0.10 (ns)	0.38 (ns)	0.006 (**)
**Fg-infected tissues (year-to-year)**				
b. Fg-infected base 2016 vs Fg-infected base 2017	0.0002 (***)	0.00019 (***)	0.0007 (***)	0.00006 (****)

****(≤0.0001), ***(≤ 0.001), **(≤ 0.01), *(≤ 0.05), ns (>0.05)

Table S5. Summary of beta diversity estimations across year-to-year trials

Comparison groups	Distance matrix	PERMANOVA test	PERMDISP results	Interpretation
**a. Testing the impact of climate variability on healthy and Fg-infected transmitting silk tissues, separately.**				
a.1 Healthy silk tip (2016 vs 2017)	BC	0.001 (***)	0.88 (ns)	Seasonal effect
	UWUF	0.007 (**)	0.14 (ns)	Seasonal effect
	WUF	0.041 (*)	0.65 (ns)	Seasonal effect
a.2 Fg-infected silk tip (2016 vs 2017)	BC	0.001 (***)	0.13 (ns)	Seasonal effect
	UWUF	0.001 (***)	0.55 (ns)	Seasonal effect
	WUF	0.001 (***)	0.26 (ns)	Seasonal effect
a.3 Healthy silk base (2016 vs 2017)	BC	0.001 (***)	0.037 (*)	Either between or within group variation
	UWUF	0.001 (***)	0.143 (ns)	Seasonal effect
	WUF	0.001 (***)	0.001 (***)	Either between or within group variation
a.4 Fg-infected silk base (2016 vs 2017)	BC	0.001 (***)	0.62 (ns)	Seasonal effect
	UWUF	0.002 (**)	0.011 (**)	Either between or within group variation
	WUF	0.001 (***)	0.02 (*)	Either between or within group variation
**b. Testing the impact of Fg infection on TSM**				
b.1 Healthy vs Fg-infected tip (2016)	BC	0.075 (ns)	0.023 (*)	Due to within group variation
	UWUF	0.001 (***)	0.001 (***)	Either between or within group variation
	WUF	0.028 (*)	0.217 (ns)	Due to Fg infection
b.2 Healthy vs Fg-infected tip (2017)	BC	0.002 (**)	0.018 (*)	Either between or within group variation
	UWUF	0.001 (***)	0.001 (***)	Either between or within group variation
	WUF	0.001 (***)	0.006 (*)	Either between or within group variation
b.3 Healthy vs Fg-infected base (2016)	BC	0.01 (**)	0.071 (ns)	Due to Fg infection
	UWUF	0.001 (***)	0.001 (***)	Either between or within group variation
	WUF	0.001 (***)	0.013 (*)	Either between or within group variation
b.4. Healthy vs Fg-infected base (2017)	BC	0.003 (**)	0.755 (ns)	Due to Fg infection
	UWUF	0.001 (***)	0.001 (***)	Either between or within group variation
	WUF	0.001 (***)	0.135 (ns)	Due to Fg infection

****(≤0.0001), ***(≤ 0.001), **(≤ 0.01), *(≤ 0.05), ns (>0.05)

The sampling depths for the compared sample groups a.1, a.2, a.3, a.4 were 1398, 3707, 2969, and 7306; for groups b.1, b.2, b.3, b.4 were 1133, 1023, 3248, and 2943, respectively.

Table S6. Summary of *Fg*-indicator taxa calculated for each silk tissue location and sorted by year. Category (A) represents *Fg*-indicator taxa that were consistently identified across two consecutive field years. **Category (B)** represents indicator taxa identified exclusively in one year. The DESeq2 R package was used to estimate the log2 fold change (LFC) in taxa abundance at an adjusted *p* value of <0.05.

**Taxonomy**			**Silk location**	**Year**	**LFC**	**Core TSM**
**Class**	**Family**	**Feature/OTU**				
**Category (A)**						
Gammaproteobacteria	Enterobacteriaceae	OTU2	Tip	2016	2.69	A,B,C,D,E,F,G,H
			Tip	2017	4.97	
			Base	2017	3.42	
Gammaproteobacteria	Enterobacteriaceae	OTU3	Tip	2016	1.55	A,B,C,D,E,F,G,H
			Tip	2017	2.29	
			Base	2016	1.78	
Gammaproteobacteria	Enterobacteriaceae	OTU4	Tip	2016	1.54	A,B,C,D,F,H
			Tip	2017	2.32	
			Base	2017	3.05	
Gammaproteobacteria	Xanthomonadaceae	Stenotrophomonas (OTU34)	Tip	2016	3.65	A,B,C,D,E,F,G,H
			Tip	2017	3.93	
			Base	2017	2.41	
Gammaproteobacteria	Burkholderiaceae	Herbaspirillum (OTU7)	Tip	2016	2.50	A,B,C,D,E,F,G,H
			Tip	2017	4.23	
Gammaproteobacteria	Burkholderiaceae	Delfitia (OTU8)	Tip	2016	3.43	B,D,E,F,G,H
			Tip	2017	3.05	
Alphaproteobacteria	Sphingomonadaceae	Sphingomonas (OTU35)	Tip	2016	1.61	A,B,C,D,E,F,G,H
			Tip	2017	2.54	
**Category (B)**						
Gammaproteobacteria	Enterobacteriaceae	OTU27	Tip	2016	4.18	B,D,E,F,G,H
Gammaproteobacteria	Enterobacteriaceae	OTU17	Base	2017	2.54	B,D,E,F,G,H
Gammaproteobacteria	Enterobacteriaceae	OTU5	Tip	2017	6.99	-
Gammaproteobacteria	Enterobacteriaceae	Serratia OTU26	Tip	2016	2.93	A,B,E,F,G
			Base	2016	2.05	
Gammaproteobacteria	Enterobacteriaceae	Pantoea OTU25	Tip	2017	1.89	A,B,C,D,E,F,G,H
			Base	2017	1.86	
Gammaproteobacteria	Enterobacteriaceae	OTU6	Base	2017	1.96	A,B,C,D,E,F,G,H

Gammaproteobacteria	Moraxellaceae	Acinetobacter (OTU32)	Tip	2017	2.67	B,D,F,G,H
Gammaproteobacteria	Moraxellaceae	Acinetobacter (OTU30)	Tip	2016	1.73	A,B,E,F,G
Gammaproteobacteria	Xanthomonadaceae	Stenotrophomonas (OTU33)	Tip	2016	2.45	A,B,E,F,G
			Base	2016	1.97	
Gammaproteobacteria	Xanthomonadaceae	Stenotrophomonas (OTU69)	Tip	2016	2.63	B
Gammaproteobacteria	Burkholderiaceae	OTU9	Tip	2017	4.33	D
Gammaproteobacteria	Burkholderiaceae	OTU24	Tip	2016	3.88	F
Gammaproteobacteria	Pseudomonadaceae	Pseudomonas (OTU50)	Tip	2017	4.15	A,B,D,G,H
Gammaproteobacteria	Rhodanobacteraceae	Luteibacter (OTU39)	Tip	2017	4.59	G,H
Alphaproteobacteria	Acetobacteraceae	OTU10	Tip	2016	2.16	A,B,E,F,G,H
Alphaproteobacteria	Rhizobiaceae	Rhizobium (OTU49)	Tip	2017	4.26	-
Bacteroidia	Sphingobacteriaceae	Sphingobacterium (OTU45)	Tip	2017	3.24	B,D,G,H
Bacteroidia	Sphingobacteriaceae	Pedobacter (OTU46)	Tip	2016	2.87	B,D,E,F

Abbreviation: LFC - Log2 fold change

Sample groups:

A. Healthy tip 2016

B. Fg-infected tip 2016

C. Healthy tip 2017

D. Fg -infected tip 2017

E. Healthy base 2016

F. Fg-infected base 2016

G. Healthy base 2017

H. Fg-infected base 2017

Supplementary Table S7. Summary of the bioinformatically predicted TSM metabolic pathways that significantly changed upon *Fg* infection. Detailed description of the predicted metabolic pathways that exhibited a significant shift/change in their activity upon Fg infection in: (a) silk tip/base tissues consistently across two-years of field trials, and in (b) a single silk tissue or year.

Pathway code	Pathway description	silk tissue/year	Expected taxonomic range	SUPERCLASSES
**Category (a)**				
BENZCOA-PWY	anaerobic aromatic compound degradation (Thauera aromatica)	Tip2016	Proteobacteria	Degradation/Utilization/Assimilation → Aromatic Compound Degradation
		Tip2017		
		Base2017		
ILEUDEG-PWY	L-isoleucine degradation I	Tip2016	Archaea, Bacteria , Eukaryota	Degradation/Utilization/Assimilation → Amino Acid Degradation → Proteinogenic Amino Acid Degradation → L-isoleucine Degradation
		Tip2017		
		Base2016		
PWY-5737	carbapenem carboxylate biosynthesis	Tip2016	Bacteria	Biosynthesis → Secondary Metabolite Biosynthesis → Antibiotic Biosynthesis
		Tip2017		
PWY-6919	neopentalenoketolactone and pentalenate biosynthesis	Tip2016	Streptomyces	Biosynthesis → Secondary Metabolite Biosynthesis → Antibiotic Biosynthesis
		Tip2017		
		Base2017		
PWY-7014	paromamine biosynthesis I	Tip2016	Actinobacteria	Biosynthesis → Secondary Metabolite Biosynthesis → Antibiotic Biosynthesis → Paromamine Biosynthesis
		Tip2017		
PWY-7031	protein N-glycosylation (bacterial)	Tip2016	Campylobacter	Biosynthesis → Carbohydrate Biosynthesis → Glycan Biosynthesis → Protein Glycosylation<br>Biosynthesis → Carbohydrate Biosynthesis → Oligosaccharide Biosynthesis<br>Glycan Pathways → Glycan Biosynthesis → Protein Glycosylation<br>Macromolecule Modification → Protein Modification → Protein Glycosylation
		Tip2017		
PWY-7274	D-cycloserine biosynthesis	Tip2016	Streptomycetaceae	Biosynthesis → Amino Acid Biosynthesis → Other Amino Acid Biosynthesis<br>Biosynthesis → Secondary Metabolite Biosynthesis → Antibiotic Biosynthesis
		Tip2017		
PWY-7316	dTDP-N-acetylviosamine biosynthesis	Tip2016	Bacteria	Biosynthesis → Carbohydrate Biosynthesis → Sugar Biosynthesis → Sugar Nucleotide Biosynthesis → dTDP-sugar Biosynthesis
		Tip2017		
		Base2017		
PWY-7401	crotonate fermentation (to acetate and cyclohexane carboxylate)	Tip2016	Bacteria	Generation of Precursor Metabolite and Energy → Fermentation<br>Superpathways
		Tip2017		
PWY-6145	superpathway of CMP-sialic acids biosynthesis	Tip2017	Bacteria , Eukaryota	Biosynthesis → Carbohydrate Biosynthesis → Sugar Biosynthesis → Sugar Nucleotide Biosynthesis → CMP-sugar Biosynthesis<br>Superpathways
		Base2016		
		Base2017		
PWY-7413	dTDP-6-deoxy-α-D-allose biosynthesis	Base2016	Bacteria	Biosynthesis → Carbohydrate Biosynthesis → Sugar Biosynthesis → Sugar Nucleotide Biosynthesis → dTDP-sugar Biosynthesis
		Base2017		
**Category (b)**				
PWY-6395	superpathway of seleno-compound metabolism	Tip2016	Bacteria , Fungi, Viridiplantae	Biosynthesis → Amino Acid Biosynthesis → Other Amino Acid Biosynthesis
		Base2016		Degradation/Utilization/Assimilation → Inorganic Nutrient Metabolism → Selenium Metabolism → Seleno-Amino Acid Detoxification
				Detoxification → Seleno-Amino Acid Detoxification Superpathways
PWY-6141	archaetidylserine and archaetidylethanolamine biosynthesis	Tip2017	Archaea	Biosynthesis → Fatty Acid and Lipid Biosynthesis → Phospholipid Biosynthesis<br>Superpathways
		Base2017		
PWY-6942	dTDP-D-desosamine biosynthesis	Tip2017	Bacteria	Biosynthesis → Carbohydrate Biosynthesis → Sugar Biosynthesis → Sugar Nucleotide Biosynthesis → dTDP-sugar Biosynthesis
		Base2017		
separator				
PWY-2201	folate transformations I	Tip2016	Archaea, Bacteria <bacteria>, Eukaryota	Biosynthesis → Cofactor, Prosthetic Group, Electron Carrier, and Vitamin Biosynthesis → Vitamin Biosynthesis → Folate Biosynthesis → Folate Transformations
PWY-5184	toluene degradation VI (anaerobic)	Tip2016	Proteobacteria	Degradation/Utilization/Assimilation → Aromatic Compound Degradation → Toluene Degradation<br>Superpathways
PWY-5519	D-arabinose degradation III	Tip2017	Thermoprotei	Degradation/Utilization/Assimilation → Carbohydrate Degradation → Sugar Degradation → D-arabinose Degradation
PWY-5757	fosfomycin biosynthesis	Tip2016	Bacteria <bacteria>	Biosynthesis → Secondary Metabolite Biosynthesis → Antibiotic Biosynthesis
PWY-5789	3-hydroxypropanoate/4-hydroxybutanate cycle	Tip2016	Crenarchaeota <Crenarchaeota>	Degradation/Utilization/Assimilation → C1 Compound Utilization and Assimilation → CO2 Fixation → Autotrophic CO2 Fixation
PWY-5940	streptomycin biosynthesis	Tip2017	Actinobacteria <actinobacteria>	Biosynthesis → Secondary Metabolite Biosynthesis → Antibiotic Biosynthesis
PWY-6143	CMP-pseudaminate biosynthesis	Tip2017	Bacteria <bacteria>	Biosynthesis → Carbohydrate Biosynthesis → Sugar Biosynthesis → Sugar Nucleotide Biosynthesis → CMP-sugar Biosynthesis
PWY-6146	Methanobacterium thermoautotrophicum biosynthetic metabolism	Tip2016	Archaea	Biosynthesis<br>Superpathways
PWY-6281	L-selenocysteine biosynthesis II (archaea and eukaryotes)	Tip2016	Archaea, Eukaryota	Biosynthesis → Amino Acid Biosynthesis → Proteinogenic Amino Acid Biosynthesis → L-selenocysteine Biosynthesis
PWY-6565	superpathway of polyamine biosynthesis III	Tip2017	Vibrionaceae	Biosynthesis → Amine and Polyamine Biosynthesis<br>Superpathways
PWY-6760	D-xylose degradation III	Tip2017	Archaea	Degradation/Utilization/Assimilation → Carbohydrate Degradation → Sugar Degradation → Xylose Degradation
PWY-6797	6-hydroxymethyl-dihydropterin diphosphate biosynthesis II (Methanocaldococcus)	Tip2017	Archaea	Biosynthesis → Cofactor, Prosthetic Group, Electron Carrier, and Vitamin Biosynthesis → Vitamin Biosynthesis → Folate Biosynthesis →
				6-Hydroxymethyl-Dihydropterin Diphosphate Biosynthesis
PWY-6975	superpathway of erythromycin biosynthesis (without sugar biosynthesis)	Tip2016	Bacteria <bacteria>	Biosynthesis → Secondary Metabolite Biosynthesis → Antibiotic Biosynthesis → Macrolide Antibiotic Biosynthesis<br>Superpathways
PWY-6977	superpathway of erythromycin biosynthesis	Tip2016		
PWY-6993	nicotine degradation II (pyrrolidine pathway)	Tip2016	Bacteria <bacteria>	Degradation/Utilization/Assimilation → Degradation/Utilization/Assimilation - Other → Nicotine Degradation
PWY-7002	4-hydroxyacetophenone degradation	Tip2017	Bacteria <bacteria>	Degradation/Utilization/Assimilation → Aromatic Compound Degradation
PWY-7015	ribostamycin biosynthesis	Tip2016	Bacteria <bacteria>	Biosynthesis → Secondary Metabolite Biosynthesis → Antibiotic Biosynthesis
PWY-7019	butirosin biosynthesis	Tip2016	Bacteria <bacteria>	Biosynthesis → Secondary Metabolite Biosynthesis → Antibiotic Biosynthesis
PWY-7020	superpathway of butirocin biosynthesis	Tip2016	Bacteria <bacteria>	Biosynthesis → Secondary Metabolite Biosynthesis → Antibiotic Biosynthesis<br>Superpathways
PWY-7022	paromamine biosynthesis II	Tip2016	Bacteria <bacteria>	Biosynthesis → Secondary Metabolite Biosynthesis → Antibiotic Biosynthesis → Paromamine Biosynthesis
		Base2017		

PWY-7106	erythromycin D biosynthesis	Tip2016	Bacteria <bacteria>	Biosynthesis → Secondary Metabolite Biosynthesis → Antibiotic Biosynthesis → Macrolide Antibiotic Biosynthesis
PWY-7110	superpathway of megalomicin A biosynthesis	Tip2016	Bacteria <bacteria>	Biosynthesis → Secondary Metabolite Biosynthesis → Antibiotic Biosynthesis → Macrolide Antibiotic Biosynthesis<br>Superpathways
PWY-7287	novobiocin biosynthesis	Tip2016	Actinobacteria <actinobacteria>	Biosynthesis → Secondary Metabolite Biosynthesis → Antibiotic Biosynthesis<br>Superpathways
PWY-7352	daunorubicin biosynthesis	Tip2017	Streptomycetaceae	Biosynthesis → Secondary Metabolite Biosynthesis → Antibiotic Biosynthesis
PWY-7354	aclacinomycin biosynthesis	Tip2017	Streptomycetaceae	Biosynthesis → Secondary Metabolite Biosynthesis → Antibiotic Biosynthesis
PWY-7402	benzoate fermentation (to acetate and cyclohexane carboxylate)	Tip2016	Bacteria <bacteria>	Generation of Precursor Metabolite and Energy → Fermentation<br>Superpathways
		Base2017		
NPGLUCAT-PWY	Entner-Doudoroff pathway II (non-phosphorylative)	Base2017	Thermoplasmata, Thermoprotei	Degradation/Utilization/Assimilation → Carbohydrate Degradation → Sugar Degradation → Entner-Duodoroff Pathways<br>Generation of Precursor Metabolite and Energy → Entner-Duodoroff Pathways
PWY-5427	naphthalene degradation (aerobic)	Base2017	Bacteria <bacteria>	Degradation/Utilization/Assimilation → Aromatic Compound Degradation → Naphthalene Degradation
PWY-6946	cholesterol degradation to androstenedione II (cholesterol dehydrogenase)	Base2017	Bacteria <bacteria>	Degradation/Utilization/Assimilation → Fatty Acid and Lipid Degradation → Steroid Degradation → Cholesterol Degradation
PWY-7415	tylosin biosynthesis	Base2017	Streptomyces	Biosynthesis → Secondary Metabolite Biosynthesis → Antibiotic Biosynthesis → Macrolide Antibiotic Biosynthesis
PWY-7524	mevalonate pathway III (archaea)	Base2017	Archaea	Biosynthesis → Secondary Metabolite Biosynthesis → Terpenoid Biosynthesis → Hemiterpene Biosynthesis → Isopentenyl Diphosphate Biosynthesis<br>→ Mevalonate Pathways
